# Evaluation of candidate vaccine approaches for MERS-CoV

**DOI:** 10.1038/ncomms8712

**Published:** 2015-07-28

**Authors:** Lingshu Wang, Wei Shi, M. Gordon Joyce, Kayvon Modjarrad, Yi Zhang, Kwanyee Leung, Christopher R. Lees, Tongqing Zhou, Hadi M. Yassine, Masaru Kanekiyo, Zhi-yong Yang, Xuejun Chen, Michelle M. Becker, Megan Freeman, Leatrice Vogel, Joshua C. Johnson, Gene Olinger, John P. Todd, Ulas Bagci, Jeffrey Solomon, Daniel J. Mollura, Lisa Hensley, Peter Jahrling, Mark R. Denison, Srinivas S. Rao, Kanta Subbarao, Peter D. Kwong, John R. Mascola, Wing-Pui Kong, Barney S. Graham

**Affiliations:** 1 Vaccine Research Center, National Institute of Allergy and Infectious Diseases, National Institutes of Health, Bethesda, 20892 Maryland USA; 2 U.S. Military HIV Research Program, Walter Reed Army Institute of Research, Silver Spring, 20910 Maryland USA; 3 Henry M. Jackson Foundation for the Advancement of Military Medicine, Bethesda, Maryland 20817 USA; 4 Sanofi-Aventis, 270 Albany Street, Cambridge, 02139 Massachusetts USA; 5 Department of Pediatrics, Vanderbilt University Medical Center, Nashville, 37232 Tennessee USA; 6 Emerging Respiratory Viruses Section, Laboratory of Infectious Diseases, National Institute of Allergy and Infectious Diseases, National Institutes of Health, Bethesda, 20892 Maryland USA; 7 Integrated Research Facility, National Institute of Allergy and Infectious Diseases, National Institutes of Health, Frederick, 21702 Maryland USA; 8 Department of Radiology and Imaging Sciences, Center for Infectious Disease Imaging, National Institutes of Health, Bethesda, 20892 Maryland USA; 9 Center for Research in Computer Vision (CRCV), University of Central Florida, Orlando, 32816 Florida USA; 10 Department of Pathology, Microbiology and Immunology, Vanderbilt University Medical Center, Nashville, 37232 Tennessee USA

**Keywords:** Antibodies, Vaccines, Viral infection, Virology

## Abstract

The emergence of Middle East respiratory syndrome coronavirus (MERS-CoV) as a cause of severe respiratory disease highlights the need for effective approaches to CoV vaccine development. Efforts focused solely on the receptor-binding domain (RBD) of the viral Spike (S) glycoprotein may not optimize neutralizing antibody (NAb) responses. Here we show that immunogens based on full-length S DNA and S1 subunit protein elicit robust serum-neutralizing activity against several MERS-CoV strains in mice and non-human primates. Serological analysis and isolation of murine monoclonal antibodies revealed that immunization elicits NAbs to RBD and, non-RBD portions of S1 and S2 subunit. Multiple neutralization mechanisms were demonstrated by solving the atomic structure of a NAb-RBD complex, through sequencing of neutralization escape viruses and by constructing MERS-CoV S variants for serological assays. Immunization of rhesus macaques confers protection against MERS-CoV-induced radiographic pneumonia, as assessed using computerized tomography, supporting this strategy as a promising approach for MERS-CoV vaccine development.

**Supplementary information:**

The online version of this article (doi:10.1038/ncomms8712) contains supplementary material, which is available to authorized users.

## Abstract (web summary)

Unmet need exists for a vaccine against Middle East respiratory syndrome coronavirus (MERS-CoV). Here the authors report the establishment and evaluation, in mice and primates, of a series of MERS-CoV immunogens and show that they can serve as promising leads for vaccine development.

**Supplementary information:**

The online version of this article (doi:10.1038/ncomms8712) contains supplementary material, which is available to authorized users.

## Graphical abstract

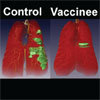

## Introduction

Middle East respiratory syndrome coronavirus (MERS-CoV) has emerged as a highly fatal cause of severe acute respiratory infection. Since April 2012, 1,348 cases and 479 deaths in over twenty-five countries have been attributed to this novel beta-coronavirus^1,2^. As human-to-human transmission of the virus is not sustained, a large zoonotic reservoir may serve as a principal source for transmission events^3,4,5,6^. The high case fatality rate, vaguely defined epidemiology, and absence of prophylactic or therapeutic measures against this novel virus have created an urgent need for an effective vaccine should the outbreak expand to pandemic proportions.

Past efforts to develop coronavirus vaccines have used whole-inactivated virus, live-attenuated virus, recombinant protein subunit or genetic approaches^7^. The primary target for neutralizing antibodies is the Spike (S) glycoprotein, cleaved into two subunits: S1, which is distal to the virus membrane and S2, which contains both a transmembrane domain and two heptad-repeat sequences typical of class I fusion glycoproteins^8,9^. The S1 subunit has been the focus of most immunization strategies against MERS-CoV^10,11,12^, as it contains the receptor-binding domain (RBD) that mediates virus attachment to its host receptor, dipeptidyl peptidase-4 (DPP4)^13^. Expressing the RBD on multiple vaccine platforms can elicit neutralizing antibodies of high potency^14,15,16,17,18^ that prevent viral attachment across many strains but will not elicit antibodies that contribute to neutralization through fusion inhibition. We developed an alternative vaccine regimen, based on full-length S DNA and a truncated S1 subunit glycoprotein, to elicit a broad repertoire of antibodies with diverse mechanisms of viral neutralization, and found that immunization with these constructs protected non-human primates (NHPs) from severe lung disease after intratracheal challenge with MERS-CoV.

## Results

### Spike glycoprotein immunogen construction and characterization

**Figure 1:**
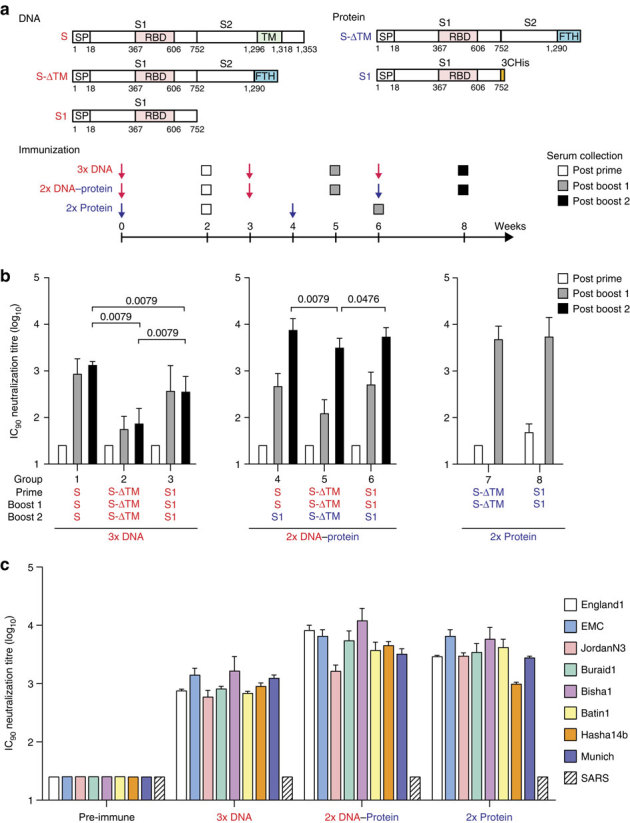
MERS-CoV Spike glycoprotein vaccine design and immunogenicity in mice. Candidate vaccine immunogens were designed on the basis of the Spike glycoprotein sequence of the England1 MERS coronavirus and elicited high neutralization titres. (**a**) Schematic representation of MERS-CoV Spike protein cDNAs and recombinant proteins. Five vaccine constructs were made: three DNA and two protein subunits. DNA constructs consisted of full-length Spike or truncated versions that either had the transmembrane domain or the entire S2 subunit deleted. The protein constructs contain either a truncated Spike molecule with the transmembrane domain deleted (S-ΔTM) or the S1 subunit. RBD, receptor-binding domain; SP, signal peptide; TM, transmembrane domain; FTH, foldon (trimerization domain), thrombin (cleavage site) followed by histidine tag; 3CHis, Human rhinovirus 3C protease cleavage site, followed by 6 × histidine tag. (**b**) Immunogenicity of eight vaccine regimens. Five mice per group were immunized with plasmid DNA only at weeks 0, 3 and 6 (groups 1–3); plasmid DNA at weeks 0 and 3 and protein plus Ribi adjuvant at week 6 (groups 4–6); or protein plus Ribi adjuvant at weeks 0 and 4 (groups 7 and 8). Two weeks after each immunization, sera were collected and neutralizing antibody titres were measured against pseudotyped MERS-CoV England1 virus. Open, grey and black bars, respectively, represent the IC_90_ neutralization titres (GMT from five mice per group with 95% confidence interval) from the post-prime, first post-boost and second post-boost sera. Each sample was tested in triplicate; all assays were repeated once. A nonparametric two-tailed *t*-test (Mann–Whitney) was used for statistical analysis, and the relevant *P* values are indicated. (**c**) MERS-CoV vaccines induced cross-neutralization to eight MERS-CoV strains. The sera from the mice immunized with MERS-CoV S DNA three times, primed with S DNA and boosted with S1 protein plus Ribi adjuvant, or primed and boosted with S1 protein plus Ribi adjuvant were assayed for neutralization to the eight strains of MERS-CoV and SARS-CoV pseudotyped viruses as indicated. IC_90_ titre is shown. Data are presented as the mean of triplicates with s.e.’s.

We originally designed five vaccine constructs on the basis of sequences from the MERS-CoV Spike glycoprotein (Fig. 1a). The England1 strain (GenBank ID: AFY13307) was chosen on the basis of the availability of its sequence and its proximity to a consensus among published sequences, particularly within the RBD. We constructed three plasmid vaccines that encoded (1) full-length, membrane-anchored Spike; (2) transmembrane-deleted (ΔTM) Spike containing the entire ectodomain; and (3) S1 subunit only. All three plasmids were delivered intramuscularly by needle and syringe, followed by electroporation. The two protein subunit vaccines included S-ΔTM and S1 and were delivered intramuscularly by needle and syringe with Ribi adjuvant. These five candidate vaccines were systematically evaluated in mice according to eight immunization regimens (Fig. 1a). To test the immunogenicity of our vaccine candidates against multiple MERS-CoV strains—without the requirement of a biosafety level 3 facility—we developed a pseudotyped reporter virus neutralization assay, as we did previously for SARS-CoV^19,20,21,22^. We confirmed that the assay measured viral entry via the MERS-CoV receptor, DPP4, by demonstrating that HEK 293 cells required DPP4 expression on their surface for efficient infection and that soluble DPP4 or anti-DPP4 antibody prevented infection (Supplementary Fig. 1a–d).

### Full-length S DNA and S1 protein elicit high titers of NAb in mice

Mice primed either once with S1 protein or twice with S DNA and then boosted once with S1 protein generated the highest neutralizing antibody (NAb) titres among all groups (Fig. 1b). The full-length S DNA regimen induced a significantly higher antibody response than the truncated S-ΔTM or S1 DNA regimens. Antibody titres tended to be either low or undetectable after the first dose of DNA but were boosted 10-fold after immunization with S1 protein. Two doses of S1 protein achieved an IC_90_ neutralization titer (90% inhibitory concentration) near that of the DNA/protein regimen. Three of the eight vaccine regimens— S DNA, S DNA-S1 protein and S1 protein alone—were carried forward for detailed evaluation. S-ΔTM gave low production yields from transfected HEK 293 cells and was not evaluated further. Immune sera (5 weeks after final boost) from these three regimens were tested against a panel of eight pseudoviruses and found to generate equally robust NAb titres against all strains (Fig. 1c). The sequence homology across strains (Supplementary Fig. 2) likely accounted for the breadth of neutralization. SARS-CoV pseudovirus was not neutralized by sera from any of the immunized mice. Pseudovirus neutralization results were compared with a live virus microneutralization assay for the JordanN3 strain (GenBank ID: KC776174.1). The pseudovirus neutralization assay was ∼10-fold more sensitive to neutralization than the live virus, although the two assays correlated well with one another on the basis of relative magnitude (Supplementary Fig. 3).

### Neutralizing antibodies to epitopes on different Spike domains

**Figure 2:**
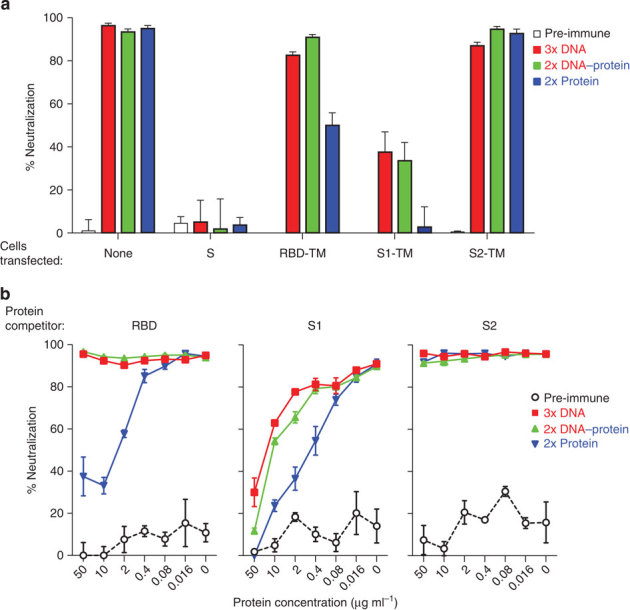
Varied vaccination regimens elicit neutralizing antibodies that target different regions of the MERS-CoV Spike glycoprotein. Immunization with different vaccine regimens elicited neutralizing antibodies that target the Spike glycoprotein within and outside the RBD. (**a**) Cell adsorption assay. Sera from mice immunized with MERS-CoV S DNA-only, S DNA prime and S1 protein plus Ribi adjuvant, or S1 protein plus Ribi adjuvant prime and boost were evaluated for neutralization activity against pseudotyped MERS-CoV England1 after adsorption with HEK 293T cell-surface-expressed MERS-CoV Spike proteins: S, RBD, S1, S2. Serum neutralization was tested at a single dilution. Sera adsorbed with untransfected HEK 293T cells served as controls and retained 95% of neutralization activity. Each bar represents the mean of triplicate assays with s.e.’s. The experiment was repeated once to ensure reproducible results; one of the two experiments is shown. (**b**) Protein competition neutralization assay. Sera at a single dilution from the immunized mice were also assayed for neutralization of MERS-CoV England1 pseudovirus in the presence of soluble MERS-CoV RBD, S1 and S2 proteins at concentrations of 0.016–50 μg ml^−1^. Each data point represents the mean of triplicate assays with s.e.’s. The experiment was repeated once to ensure reproducible results; one of the two experiments is shown.

We analysed the specificity of NAb responses against the different subunits of the MERS-CoV Spike glycoprotein using several methods. First, sera were pre-adsorbed with HEK 293T cells expressing transmembrane-anchored versions of S, S1, S2 and RBD (Fig. 1a and Supplementary Fig. 4a) and were screened for neutralization (Fig. 2a). Sera pre-adsorbed with untransfected cells retained 95% of their original neutralization activity, while negative control pre-immune sera showed no capacity for virus neutralization. The full-length Spike-transfected cells adsorbed virtually all neutralization activity from the immune sera of all three vaccine groups, while the RBD expressed by itself on the cell membrane only adsorbed about half of the neutralizing activity, and only in the S1 protein group, suggesting the possibility that the conformation of RBD presented in the full-length Spike and in the truncated versions may differ. Adsorption with S1 also removed all neutralization activities in the S1 protein group and ∼60% of neutralizing capacity in the two DNA-primed groups. In contrast, S2 did not deplete neutralization activity in any of the groups, except slightly in mice vaccinated with DNA alone. It is likely that expression of S2 by itself on cells resulted in rearrangement to the post-fusion conformation, and therefore neutralizing epitopes present on the prefusion structure would not have been available for adsorption. Flow cytometric analysis of serum bound to cells that expressed different Spike subunits gave consistent results (Supplementary Fig. 4c).

Protein competition neutralization assays recapitulated the findings of the Spike-transfected cell adsorption assays (Fig. 2b). Competition with >2 μg ml^−1^ soluble RBD (Supplementary Fig. 4b) reduced immune sera neutralization activity by ∼50–60% in the S1 protein vaccination group. As seen in the cell adsorption assay, soluble S1 protein removed neutralizing activity of sera from all three vaccine groups, although to a higher degree in the mice immunized only with S1 protein compared with the groups primed with S DNA. Soluble S2 (Supplementary Fig. 4b) had no impact on serum neutralization capacity and full-length Spike was not used as a competitor as it could not be expressed in the soluble form. Overall, these data indicated that the serum neutralization activity in the protein-only immunization group was directed primarily against the RBD. The S DNA-only or S DNA-S1 protein-induced immune sera were more complex as more than half of the neutralizing activity was directed against S1; however, there was residual activity that was not absorbed or competed by S1. It is not conclusively known why S2 did not adsorb or compete with these sera; however, it is likely that the expressed S2 protein was in a post-fusion conformation as stated above.

### Vaccine regimens elicit antibodies of different IgG subclass

Sera were also analysed for the quality of the humoral response induced by the different immunization regimens and found to elicit different IgG subclass response patterns (Supplementary Fig. 5a,b). S DNA immunization induced an IgG2a-dominant response (geometric mean titre (GMT) IgG2a/IgG1 ratio of 6), while S1 protein immunization generated an IgG1-dominant response (GMT IgG2a/IgG1 ratio of 0.06). This IgG subclass response pattern appeared to be determined by the priming immunization as the S DNA prime-S1 protein boost regimen induced a similar pattern and magnitude of IgG2a polarization as the S DNA-only regimen. The IgG2a and IgG1 subclass responses, respectively, reflect T helper 1 cell (Th1)- and T helper 2 cell (Th2)-biased immune response patterns in mice^23^. The IgG2a or Th1-biased response elicited by the DNA-containing regimens is likely due to the induction of interferon-γ-producing CD8 T cells that modulate CD4 T-cell differentiation^24,25^. The monophosphoryl lipid A-based Ribi adjuvant was not sufficient to influence the IgG subclass polarization towards a Th1 phenotype as would be expected^26,27^. Thus, compared with priming with a protein, DNA priming generated a T helper response that is generally associated with more effective control of viral infections.

### Mouse monoclonal antibody functional characterization

**Figure 3:**
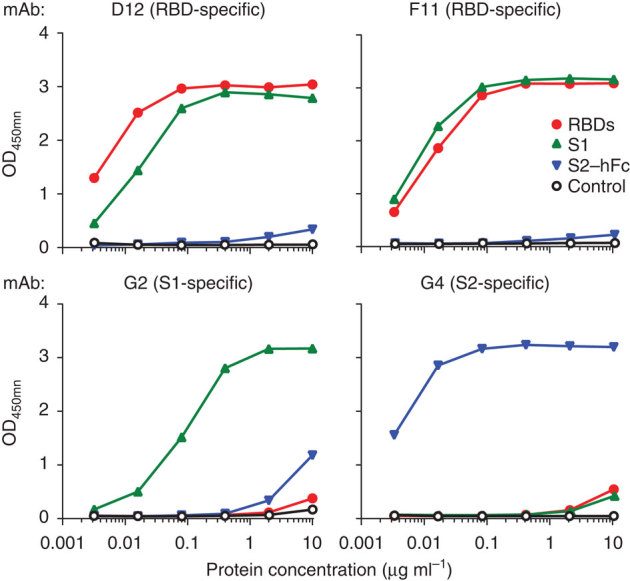
Isolated mAbs exhibit high binding affinity against multiple Spike glycoprotein (Spike) domains. Four mAbs were characterized for their binding specificity. Each of the mAbs was tested, using ELISA, in triplicate, for binding to soluble RBD, S1 and S2 conjugated to Fc (S2–hFc) respectively.

We sought to further investigate the humoral response to the S DNA-S1 protein vaccine by isolating and characterizing monoclonal antibodies (mAbs) of different specificities. Hybridomas were generated from S DNA-primed and S1 protein-boosted mice and were screened for binding to the S1, RBD and S2 domains. The final round of hybridoma screening generated 45 subclones (Supplementary Fig. 6), four of which (D12, F11, G2 and G4) were selected for additional characterization on the basis of their binding specificity and neutralization potency (Fig. 3, Table 1 and Supplementary Fig. 7). All four mAbs demonstrated, using immunofluorescence (IF), binding to live virus (EMC strain)-infected cells (Supplementary Fig. 8). D12 and F11 bound the RBD within the S1 subunit. The mAb G2 was also specific for S1; however, it bound outside the RBD. G4 bound only to the S2 subunit (Fig. 3). Although not as potent as the RBD-specific mAbs (Table 1), G2 and G4 were unique as compared with other reported mAbs against MERS-CoV^14,15,16,17^ in their ability to neutralize virus despite targeting epitopes outside the RBD. Soluble protein competition neutralization results were consistent with the binding assays result (Supplementary Fig. 9a). D12 and F11 neutralizations were diminished in the presence of RBD and S1, while G2 was competed by S1 and G4-neutralizing activity was only abrogated by high concentrations of S2.

**Table 1:** mAb-binding affinity and neutralization*

**Antibody**	**Antigen**	**Binding kinetics**			**Neutralization (μg ml**^−1^)	
		***K***_**on**_**(Ms**^−1^)	***K***_**off**_**(s**^−1^)	***K***_**D**_ **(M)**	**IC**_**50**_	**IC**_**80**_
D12	RBD	4.91E+05	4.88E−03	9.93E−09	0.013	0.04
	S1	1.15E+05	7.60E−04	6.63E−09		
F11	RBD	6.91E+04	7.90E−03	1.14E−07	0.008	0.052
	S1	1.12E+05	3.91E−04	3.49E−09		
G2	S1	1.11E+05	1.88E−04	1.69E−09	0.013	0.35
G4	S2	3.69E+04	3.19E−04	8.65E−09	0.133	0.806

mAb, monoclonal antibody; MERS-CoV, Middle East respiratory syndrome coronavirus; RBD, receptor-binding domain.

The mAbs specific for the MERS-CoV RBD—D12 and F11—demonstrated the highest neutralization potency of the four characterized mAbs. G2, which bound S1 outside the RBD, had weaker affinity to S1 than D12 and F11 but near equal neutralization potency. The S2-binding mAb, G4, had a tenfold lower neutralizing potency than the other mAbs. All experiments for this figure were repeated once to ensure reproducibility.

^*^Binding affinity and neutralization activity were measured in triplicate by biolayer interferometry (Supplementary Fig. 7) and a pseudotyped MERS-CoV (England1) virus neutralization assay, respectively.

**Figure 4:**
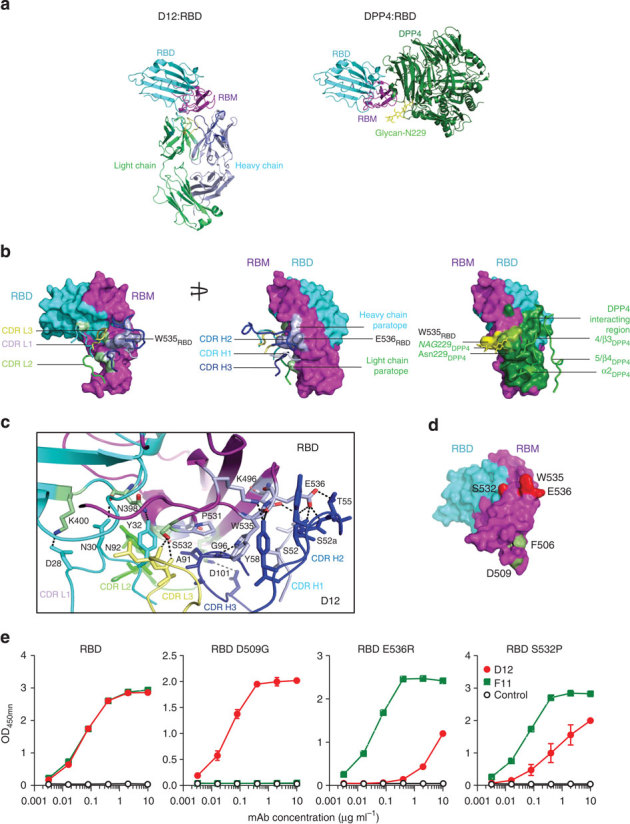
MERS-CoV-neutralizing mAbs target multiple regions of the RBD. Vaccine-induced mAb D12 binds to the DPP4-interacting region of the viral Spike RBD, blocking receptor binding. (**a**) (Left) Comparison of RBD binding to D12 and DPP4. RBD (cyan) with receptor-binding motif (residues 484–567, magenta) and D12 are shown in ribbon representation. The D12 heavy chain is light blue and the light chain is light green. The heavy chain complementarity determining regions (CDR) are light blue (CDR H1), blue (CDR H2) and dark blue (CDR H3), while the light chain CDRs are cyan (CDR L2), green (CDR L2) and pale yellow (CDR L3). The main interacting regions are in CDR H2, CDR H3 and CDR L2. (Right) DPP4 is shown in ribbon representation (green) with Asn 229 and the attached *N*-glycan (yellow) shown as sticks. The RBD is oriented as shown in the left panel. (**b**) Antibody:RBD and DPP4:RBD crystal structure complexes. RBD in surface representation is shown with the D12 heavy and light chain binding region coloured blue and green, respectively. The CDR loops are shown as ribbons and coloured as in (**a**; left). The rotated RBD shows the full D12 paratope: D12 CDR H2 interacts with RBD W535 and E536 residues, which predominantly interact with the Asn 229-associated *N*-glycan on DPP4 (centre). RBD, shown in surface representation with the DPP4-interacting region coloured green. Major interacting regions of DPP4 are shown as ribbon representations with Asn 229 and *N*-glycan shown as sticks (Supplementary Fig S11; right). (**c**) D12 and RBD interface. All CDRs are shown in ribbon representation, with interacting residues shown as sticks, and hydrogen bonds represented by dotted lines. (**d**,**e**) Crystal structure of MERS England1 RBD and effect of critical RBD mutations on binding. RBD residues 506 and 509, identified by mutagenesis analysis, are highlighted in green. Critical RBD residues 532, 535 and 536, identified by structural definition and viral resistance evolution, reduce or eliminate D12 binding (shown in red). ELISA results show that this set of mutations eliminate F11 or D12 binding.

Although the two most potent neutralizing mAbs—D12 and F11—targeted the RBD, their neutralization profiles were different, when mAb neutralization was tested against the panel of eight pseudotyped reporter viruses (Fig. 1c). Notably, F11 was unable to neutralize the Bisha1 strain (GenBank ID: KF600620.1) of MERS-CoV (Supplementary Fig. 9b), which differs from other strains by an aspartic acid to glycine substitution at residue 509, rendering it resistant to F11 while still susceptible to D12 neutralization. This finding was recapitulated in a pseudotyped virus neutralization assay where F11 neutralization activity against wild-type England1 was ablated with the introduction of a D509G mutation (Supplementary Fig. 9b). D12, in contrast, neutralized both viruses irrespective of the amino-acid change at position 509. In addition, the RBD 509G mutation abrogated F11 binding using enzyme-linked immunosorbent assay (ELISA) but did not affect D12 binding (Fig. 4e).

### Structural and mutational analyses of neutralizing antibodies

To provide an atomic-level understanding of neutralizing activity, the D12 antibody was crystallized as an Fab in complex with the England1 RBD (Fig. 4a,b, Supplementary Table 1 and Supplementary Fig. 10). In addition, the unbound structure of England1 RBD was determined (Fig. 4c) and found to be nearly identical to the RBD of the EMC strain (GenBank ID: JX869059.2), either by itself or in complex with DPP4 (ref. 28), as it has only one amino-acid difference (F506L) in the RBD (Supplementary Fig. 2). The D12 antibody forms direct contacts with the receptor-binding motif of the RBD, and the heavy-chain-interacting region overlaps with the contact region between MERS RBD and human DPP4 as defined in ref. 28. RBD residues W535 and E536, which bind to the conserved glycan on DPP4, are bound by the CDR H2 and CDR H3 within the D12 paratope (Fig. 4c). Mutation of both residues abrogated the ability of D12 to bind (Fig. 4d,e) and neutralize virus (Supplementary Fig. 9c). Additional virus neutralization escape analysis demonstrated that another residue within the epitope was critical for D12 neutralization activity. The EMC strain of MERS-CoV escaped neutralization by D12 when the serine at position 532 was mutated to either a proline or tryptophan. The introduction of a proline at position 531 removes two hydrogen bonds to the D12 antibody (Fig. 4c and Supplementary Table 2), while successive prolines at 531 and 532 (proline 532 is native to the RBD) are predicted to alter the side-chain orientation of adjacent residues, likely affecting W535 and E536 interactions with D12. Mutation to a bulky tryptophan side chain would cause a direct clash with the CDR L3, precluding the binding of D12 (Fig. 4c–e). Although it also targets the RBD, F11 neutralizes MERS-CoV by a different mechanism than D12. Site-directed mutagenesis and competition-binding studies indicate that F11 makes contact with the RBD on the opposing side of the binding site of D12, at and around residue 509. Binding studies also show that both D12 and F11 can bind to the RBD at the same time (Supplementary Fig. 11a–c), suggesting the potential for additive neutralization effects. The differing points of contact made by F11 and D12 on the MERS-CoV RBD are analogous to those of other mAbs specific for the SARS-CoV RBD (Supplementary Fig. 12a–d)^29,30^, suggesting convergent mechanisms of neutralization. Furthermore, these data elucidate mechanisms for virus neutralization that involve the disruption of binding between the N-terminal end of the MERS-CoV RBD and its host receptor DPP4.

### Potent and durable NAb titres in NHPs

**Figure 5:**
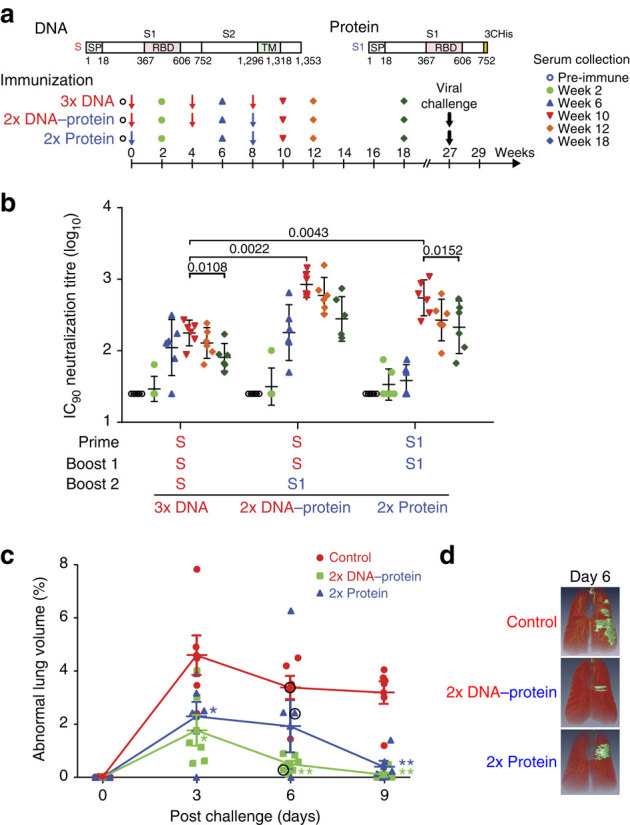
MERS-CoV Spike immunogens elicit potent, long-lived neutralization in NHPs and protect from severe lung infiltrates. Selected immunogens from mouse studies were evaluated in NHPs. (**a**) Schematic representation of full-length MERS-CoV Spike protein cDNA and recombinant S1 protein. The DNA construct consists of full-length Spike and transmembrane domain. The protein construct contains a truncated Spike with the S1 subunit. RBD, receptor-binding domain; SP, signal peptide; TM, transmembrane domain; 3CHis, Human rhinovirus 3C protease cleavage site, followed by 6 × histidine tag. (**b**) Immunogenicity of three vaccine regimens. Six NHPs, per group, were immunized intramuscularly using plasmid DNA with electroporation at weeks 0, 4 and 8; plasmid DNA with electroporation at weeks 0 and 4; and protein plus AlPO_4_ at week 8 or protein plus AlPO_4_ at weeks 0 and 8. Two weeks after immunization and at weeks 12 and 18, neutralizing antibody titres were measured against pseudotyped MERS-CoV England1. Different symbols indicate sera from six NHPs per group collected at indicated time points. IC_90_ neutralization titres (GMT with 95% confidence interval) from sera were determined. Nonparametric two-tailed *t*-test (Mann–Whitney) was used for statistical analysis. (**c**) Spike immunogens protect against pulmonary disease in NHPs. Six unimmunized NHPs and 12 NHPs were immunized with one of two selected candidate vaccine immunogens ((1) full-length S DNA prime/S1 subunit protein boost; (2) S1 subunit protein prime/S1 subunit protein boost) and challenged with MERS-CoV 19 weeks after last vaccine boost. Intratracheal inoculation of 5 × 10^6^ p.f.u. of the JordanN3 strain (GenBank ID: KC776174.1) was performed on each animal. The per cent abnormal lung volume in all NHPs peaked on day 3 post challenge; however, the lung infiltrates were significantly more extensive and prolonged in the unvaccinated compared with vaccinated NHPs. A nonparametric two-tailed *t*-test (Mann–Whitney) was used. *P value <0.05; ** *P* value<0.01. (**d**) Abnormal lung segmental images from selected animals on day 6 post challenge. The images correspond to NHP lung volume data points circled in black in Fig. 5c. The CT images and abnormal lung segments for all 18 animals are shown in Supplementary Fig. 14 a–c.

Of the eight vaccine regimens tested in mice, the three most immunogenic were taken forward for evaluation in NHPs (Fig. 5a). Comparable to the mice, NHPs primed with either S1 protein (plus aluminium phosphate (AlPO_4_) adjuvant) or S DNA (followed by electroporation) and boosted with S1 protein (plus AlPO_4_ adjuvant) generated the highest NAb titres, as measured by the pseudotyped virus neutralization assay, compared with the S DNA-only group (Fig. 5b). Both groups initially had low antibody titres after priming that increased 10- to 100-fold after boosting. IC_90_ neutralization titers after the final boost were ∼1 log_10_ higher in mice than in NHPs, which could be because of the different animal models, vaccine doses or adjuvants used in the two studies. Antibody titres remained high, however, at more than 2.5 log_10_ at 10-week post boost and persisted at higher levels in the DNA–protein group. A microneutralization assay with the MERS-CoV JordanN3 strain was compared with the pseudotyped neutralization assay and demonstrated similar results. Sera from the two groups immunized with S DNA bound all epitopes recognized by the four previously characterized murine antibodies (Supplementary Fig. 13). Sera from NHPs immunized with S1 protein alone, however, blocked mAbs targeted to the RBD (D12 and F11) and non-RBD S1 subunit (G2) but not the S2 subunit (G4).

### Reduced abnormalities on chest CT in immunized NHPs

Immunized NHPs were challenged with the JordanN3 strain of MERS-CoV at 19 weeks post boost and demonstrated earlier and diminished peak lung infiltrates compared with unvaccinated NHPs (Fig. 5c). Immunization with the S1/S1 protein or S DNA/S1 protein resulted in a respective four- to six-fold reduction in the peak proportional volume of pulmonary consolidation (Fig. 5c), as demonstrated using high-resolution computed tomography (CT, Fig. 5d and Supplementary Fig. 14a–c) and analysed by a previously tested method of lung segmentation^31^. NHPs immunized with S DNA/S1 protein experienced a lower peak volume of pulmonary disease than the S1/S1 protein group and cleared the pulmonary infiltrates more rapidly. All three groups exhibited a boost in anti-S1 IgG antibody and virus neutralization titres after challenge. A greater magnitude of rise in titres was observed in the unvaccinated group. Although virus isolates could not be consistently obtained from oral, nasopharyngeal or tracheal swabs in any of the three challenged groups, anti-S1 IgG and neutralizing antibodies were detected 2 weeks after the challenge in the unvaccinated group and were boosted in the vaccinated groups (Supplementary Fig. 15a,b), suggesting that viral antigen was produced by infection. No pre- or post-challenge differences between vaccinated and unvaccinated NHPs were observed in clinical symptoms, laboratory test values or histopathology parameters. In addition, there was no statistically significant correlation between neutralization antibodies either on the day of challenge or at peak (2 weeks after the last boost) with the percentage of abnormal lung volume (Supplementary Fig. 15c,d).

## Discussion

In summary, we developed DNA expression vectors and soluble protein immunogens that elicited cross-reactive neutralizing antibodies against known, circulating strains of MERS-CoV. Specifically, immunization with DNA expressing full-length Spike (plus electroporation) followed by the S1 subunit protein yielded potent neutralizing mAbs in both mice and NHPs. The current immunization strategy is the first to induce MERS-CoV-neutralizing antibodies that target multiple epitopes, both within and outside the RBD, which may potentially improve immunogenicity and reduce the likelihood of escape mutations. In the context of MERS-CoV, this is also the first time that a combination of DNA and protein immunogens has been tested in an animal model and the first time any immunization regimen has been evaluated and demonstrated protection in NHPs, with the caveats mentioned below.

Recently, two other animal challenge models have been reported in immunized mice. The first is of mice transduced with an adenoviral vector expressing human DPP4 (ref. 32). In this model, Zhao *et al*. were able to demonstrate productive infection in challenged mice and reduction of viral titres in the lungs of mice immunized with a Venezuela equine encephalitis replicon particles expressing the MERS-CoV Spike glycoprotein. Although representing an advance in the development of a viable animal model for MERS-CoV, transduced mice are still limited in their relevance to human infection by the inconsistent expression of the transduced protein, in this case human DPP4. A potentially more sustainable transgenic lethal mouse model reported by Agrawal *et al*. demonstrated productive, disseminated MERS-CoV infection, with most viral recovery in the lungs and brain^33^. Rhesus macaques do not demonstrate overt clinical disease; however, they develop lower respiratory tract infections^33^ with pulmonary infiltrates when challenged with MERS-CoV. Virologic sampling from the lungs of these NHPs is complicated as a bronchoalveolar lavage, although increasing the yield of virus recovery would likely confound the radiologic findings. In addition, the JordanN3 strain used to challenge the NHPs may have been less virulent than the EMC strain used in other challenge studies of rhesus macaques. Nonetheless, the pattern of pulmonary disease on high-resolution CT differed significantly between vaccinated and unvaccinated NHPs.

In mice, induced mAbs were cross-reactive against multiple MERS-CoV strains and were directed against the RBD of the Spike glycoprotein—preventing attachment—and against epitopes outside the RBD, in the S1 and S2 subunits. The co-crystal structure and analysis of escape mutations provided a mechanistic explanation for neutralization by the RBD-directed neutralizing antibodies. The RBD-specific antibodies we isolated are comparable to others reported in the literature, particularly those isolated from human antibody phage libraries^34^. However, D12 and F11 target different epitopes and are approximately a 1,000-fold more potent in their neutralization capacities than those previously reported. D12 appears to be highly novel in epitope recognition, while F11 shows some epitope overlap with the mAb 3B12 in the T512 loop region. However, the added sensitivity of 3B12 to mutations at positions 540 and 542 is not seen with F11, thus highlighting the difference in their respective epitopes.

Compared with protein alone, S DNA prime-S1 protein boost immunization yielded a more functionally diverse repertoire of neutralizing antibodies and also generated a Th1-biased immune response. The DNA-primed regimen also offered greater and earlier protection from radiographically defined pneumonia in challenged NHPs on the basis of earlier clearance of lung infiltrates, suggesting the potential contribution of effector CD8+ T cells elicited by DNA immunization. Thus, while a protein-only MERS-CoV vaccine may be the simpler approach, there are potential advantages to include DNA priming, including improvement in the potency and durability of immune responses^35^. In the context of SARS-CoV we have previously shown, in mice, that a DNA prime coupled with an inactivated virus boost enhanced both cellular and humoral immunities^36^. Through modifications in the boost interval, additional improvements in the magnitude and functional properties of the antibody response may also be achieved, as is the case for conventional influenza vaccines^37,38,39^, although further studies would be needed to confirm this for these MERS-CoV immunogens.

The presentation of the Spike trimer in a more native conformation on the surface of transduced cells after DNA immunization may have contributed to the generation of a diverse set of antibodies that could neutralize MERS-CoV by targeting the RBD and also epitopes outside the RBD, which may be more capable than RBD-specific antibodies at preventing viral escape variants^40^. In addition to providing a broader array of functional antibody responses, non-RBD-directed antibodies may aid in solving the trimeric Spike glycoprotein structure by targeting quarternary epitopes and stabilizing the prefusion conformation. Structural information on native pre-triggered glycoprotein trimers will be important for informing second-generation immunogen designs, as it has been for respiratory syncytial virus^41,42^. Experimental vaccines that yield mAbs provide information on the key viral structures and mechanisms of neutralization to guide improvements in MERS-CoV immunogens. A similar iterative cycle of rapid, empirical antigen design, antibody discovery and development of reagents and assays to characterize immunogenicity in different animal models may also be applicable to other emerging viral pathogens^42^.

## Methods

### Ethics statement

Animal experiments were carried out in compliance with all pertinent US National Institutes of Health regulations and policies. The National Institutes of Health, National Institute of Allergy and Infectious Diseases, Vaccine Research Center Animal Care and Use Committee reviewed and approved all animal experiments.

### DNA and protein vector constructs

We constructed DNA vaccines of MERS-CoV England1 strain Spike (strain England1, GenBank ID: AFY13307) and two truncated versions, S-ΔTM and S1 (Fig. 1a). Amino-acid sequences were obtained from GenBank, reverse-translated and codon-optimized for human cell expression. Seventy-five-base pair (bp) oligonucleotide sets with twenty-five-base pair overlaps were synthesized and gel-purified. Oligonucleotides were assembled into DNA fragments using the *Pfu* Turbo Hotstart DNA polymerase (Stratagene, La Jolla, CA) at a 50–65 °C gradient annealing temperature. DNA fragments were cloned into the pCR-Blunt II-Topo vector (Invitrogen, Carlsbad, CA) and were sequenced before cloning into the mammalian expression vector VRC8400 (refs 43, 44).

All other truncated and domain-swapping mutants were synthesized with PCR, using a full-length Spike template (Fig. 1a) and cloned into the sequence-confirmed VRC8400 plasmid. Proteins were expressed by transfection with vectors encoding corresponding genes in the Expi293 cell line. Transfected cell culture supernatants were collected and purified through HisTrap HP Hiload 16/60 Superdex columns (GE Healthcare, Piscataway, NJ, USA).

We synthesized cDNAs encoding Spike using the QuikChange XL kit (Stratagene) and introduced divergent amino acids into the parental Spike strain (England1) predicted from translated sequences of other strains Batin1, GenBank ID KF600628, Bisha1, GenBank ID: KF600620, Buraidah1, GenBank ID: KF600630, EMC, GenBank ID: AFS88936, Hasa14b, GenBank ID: KF600643, JordanN3, GenBank ID: KC776174 and Munich, GenBank ID: KF192507. All constructs were confirmed using sequencing.

### Cell lines and pseudovirus production

HEK 293T cells were obtained from ATCC (Manassas, VA, USA), and Huh7.5 cells were provided by Dr Deborah R. Taylor of the US FDA. Cell lines were cultured in DMEM with 10% BSA, 2 mM glutamine and 1 × penicillin/streptomycin (D10) in a 37 °C incubator containing 5% CO_2_. Overall, 15 × 10^6^ HEK 293T cells were aliquoted into a 15-cm plate and co-transfected with three plasmids (17.5 μg of packaging plasmid pCMVΔR8.2, 17.5 μg of transducing plasmid pHR’ CMV-Luc and 1 μg of CMV/R-MERS-CoV S plasmid) and calcium phosphate transfection reagent (Invitrogen)^21,22^. After overnight incubation, the medium was replaced with fresh D10. Forty-eight hours later, the supernatants were collected, filtered, aliquoted and frozen at −80 °C. Pseudovirus was titrated by first plating 1 × 10^4^ Huh7.5 cells per well in a 96-well white/black Isoplate (PerkinElmer, Waltham, MA) and culturing overnight. The medium was removed and twofold serial dilutions of pseudovirus were added to the cells. After 2 h of incubation, 100 μl of fresh media was added. Cells were lysed 72 h later and 50 μl of luciferase substrate (Promega, Madison, WI) was added to each well. Luciferase activity was measured according to relative luciferase unit (CPS, count per second) using the Microbeta luminescence counter (PerkinElmer).

### Phylogenetic analysis

Full-length amino-acid sequences of Spike from MERS-CoV strains England1, Munich, EMC, Buraidah1, Bisha1, Batin1, Hasa14b and JordanN3 were aligned using MAFFT L-INS-i (version 6.8.6.4, http://trex.uqam.ca). A phylogenetic tree was generated by the average linkage (UPGMA) method. The phylogenetic tree was drawn on an iTOL server (http://itol.embl.de).

### Mouse immunizations

Female BALB/cJ mice aged 6–8 weeks (Jackson Laboratory, Bar Harbor, ME) were immunized according to several regimens: (i) 3 × S DNA, (ii) 2 × S DNA-S1 protein and (iii) 2 × S1 protein. Within the DNA-only category (i) three groups of mice were injected with plasmid DNA encoding either MERS-CoV full-length Spike (group 1), Spike with a deleted transmembrane unit (S-ΔTM; group 2) or S1 (group 3) intramuscularly, followed by electroporation (manufacturer-recommended setting) with the AgilePulse System (Harvard Apparatus, Holliston, MA) at weeks 0, 3 and 6. The DNA–protein groups (ii) were injected twice with plasmid DNA either encoding MERS-CoV full-length Spike (group 4), S-ΔTM (group 5) or S1 (group 6) at weeks 0 and 3 as described and boosted with either MERS-CoV S-ΔTM (group 5) or S1 (groups 4 and 6) protein plus Ribi adjuvant (Sigma-Aldrich, St. Louis, MO) at week 6. The protein-only groups (iii) were injected twice with MERS-CoV S-ΔTM (group 7) or S1 protein (group 8) plus Ribi adjuvant at weeks 0 and 4.

Twenty micrograms of plasmid DNA in a total volume of 100 μl of PBS 10 μg of protein in 50 μl PBS mixed with an equal volume of Ribi adjuvant comprised the DNA and protein immunizations, respectively. Two weeks after each injection, sera were collected for measurement of antibody responses.

### NHP immunizations

Eighteen (6 female and 12 male) Indian rhesus macaques (*Macaca mulatta*) that weighed between 3.2 and 4.8 kg and had a mean age of 4.4 years were randomly assigned to the three groups according to sex and weight and were immunized according to one of the three different vaccine regimens: 3 × S DNA, 2 × S DNA-S1 protein or 2 × S1 protein. Sample size was based on convention. In the DNA-only group, six NHPs were injected with 1 mg of plasmid DNA encoding MERS-CoV full-length Spike at weeks 0, 4 and 8. Six NHPs in the S DNA-S1 protein group were injected with 1 mg of plasmid DNA encoding MERS-CoV full-length Spike at weeks 0 and 4 and were boosted with 100 μg of MERS-CoV S1 protein and AlPO_4_ adjuvant (Brenntag Biosector, Frederikssund, Denmark) at week 8. Six NHPs in the protein-only group were injected with 100 μg of MERS-CoV S1 protein and AlPO_4_ adjuvant at weeks 0 and 8. Plasmid DNA was given intramuscularly, followed by electroporation, and protein was injected intramuscularly. Sera were collected 2 weeks after each injection and every 2–4 weeks thereafter through week 18.

### NHP challenge

Eighteen Indian rhesus macaques—six unvaccinated, six vaccinated with the S DNA/S1 protein and six vaccinated with the S1/S1 protein—were challenged with the JordanN3 strain of MERS-CoV (GenBank ID: KC776174.1) in an approved high-containment facility 19 weeks after the last immunization. NHPs were inoculated intratracheally with 1 ml of 5 × 10^6^ p.f.u. via laryngoscope and lubricated catheter. Virus challenge inoculum was assayed to determine delivered dose. Delivered doses were 3.1 × 10^6^, 3.6 × 10^6^ and 3.4 × 10^6^ p.f.u. for cohorts 1, 2 and 3, respectively. All NHPs were monitored by clinical, laboratory and radiologic parameters at days 3, 6, 9 and 14 post-challenge. On day 28, all NHPs underwent necropsy.

### NHP radiologic assessment

Before and after virus challenge, NHPs underwent chest CT, with the PET/CT Gemini TOF imager (Philips, Andover, MA). CT images were evaluated and analysed by two blinded, board-certified radiologists. Lung pathology was assessed longitudinally using a published pulmonary imaging analysis technique^31^. An image segmentation method was used to determine the lung region of interest, followed by a machine-learning algorithm (Random Forest), that assessed the tissue class (that is, normal or abnormal) for each pixel within the region of interest and that quantified disease severity based on quantitative total lung capacity, pathology volume (if present) and proportion (ratio of pathology volume to total lung volume). The Random Forest algorithm was tuned according to ground glass opacity and consolidation patterns as these two patterns have constituted pathological formations in previous experiments.

### Pseudovirus neutralization assay

Huh7.5 cells (10,000 cells per well) were plated into 96-well white/black Isoplates (PerkinElmer) the day before infection. Serum serial dilutions were mixed with different strains of titrated pseudovirus, incubated for 30 min (min) at room temperature and added to Huh7.5 cells in triplicate. Following 2 h of incubation, wells were replenished with 100 μl of fresh media. Cells were lysed 72 h later and luciferase activity was measured. IC_90_ neutralization titres were calculated for each individual mouse serum sample.

### Microneutralization assay

Twofold dilutions of heat-inactivated, grouped sera were tested for the presence of antibodies that neutralized the infectivity of 100 × TCID_50_ of MERS-CoV JordanN3 strain in Vero cell monolayers, using four wells per dilution in a 96-well plate. Viral cytopathic effect was read on days 3 and 4. The serum dilution that completely prevented cytopathic effect in 50% of the wells was calculated using the Reed–Muench formula^45^.

### Cell-surface binding using FACS

HEK 293 cells were transfected with plasmids expressing MERS-CoV full-length S, RBD-HATM (Spike RBD with an influenza haemagglutinin transmembrane domain), S1-TM and S2-TM. Twenty-four hours later, cells were detached with 4 mM EDTA in PBS and stained with Vivid viability dye (Invitrogen). Cells were then stained with mouse sera (1:200 dilution). Cells were subsequently stained with goat anti-mouse IgG-Phycoerythrin (Santa Cruz Biotechnology, Santa Cruz, CA) and sorted with an LSR (BD Biosciences, San Jose, CA). Data were analysed using the FlowJo software (Tree Star Inc., Ashland, OR).

### ELISA measurement of IgG response

ELISA plates were coated with the MERS-CoV S1 protein at 1 μg ml^−1^ in PBS at 4 °C overnight. After standard washes and blocks, plates were incubated with serial dilutions of mouse sera. Anti-mouse IgG1 or IgG2a–horseradish peroxidase conjugates (Jackson Laboratory) were used as secondary antibodies, and 3,5,3′5′-tetramethylbenzidine (TMB) (KPL, Gaithersburg, MD) was used as the substrate to detect MERS-CoV S1-specific IgG1 or IgG2a antibody responses. End point titres were calculated as the highest serum dilution that gave an optical density exceeding five times the background reading.

### Microscopy

Sixty per cent confluent WHO-Vero cells on glass coverslips were infected with MERS-CoV at 1 p.f.u. per cell. At 24 hours post infection, cells were fixed and permeabilized in methanol at −20 ^o^C. These steps occurred under BSL3 conditions using protocols approved by the Institutional Biosafety Committee of Vanderbilt University and the CDC. Cells were rehydrated in PBS, blocked in PBS and 5% BSA and rinsed with IF wash (PBS with 1% BSA and 0.05% Nonidet P-40). Cells were incubated in primary antibodies at indicated dilutions for 45 min and then rinsed with IF wash for 3 × 5 min and incubated with secondary antibodies (Goat α-mouse-AlexaFluor 546 (1:1,500), Molecular Probes) for 30 min. Cells were washed for 3 × 5 min in IF wash, 1 × with PBS and then 1 × with distilled water. Coverslips were mounted with Aquapolymount (Polysciences) and visualized using IF microscopy on a Nikon Eclipse TE-2000S wide-field fluorescent microscope using × 40 oil-immersion through differential interference contrast, Cy3 and 4,6-diamidino-2-phenylindole filters. Images were merged and assembled using Nikon Elements, ImageJ and Adobe Photoshop CS2. Infected and mock-infected cells were processed in parallel.

### Hybridoma generation

Mice in groups 1 and 4 were boosted intravenously with an additional 20 μg of S1. Three days later, splenocytes were harvested and fused with Sp2/0 myeloma cells (ATCC) using polyethylene glycol (PEG) 1450 (50% (w/v), Sigma-Aldrich) according to the standard methods. Cells were cultured and screened in RPMI complete medium that contained 20% FCS and 1 × 100 μM hypoxanthine, 0.4 μM aminopterin and 16 μM thymidine (Sigma-Aldrich). Supernatants from resulting hybridomas were screened for binding, using ELISA, to MERS S1, RBD or S-ΔTM as well as for neutralizing activity. Subclones were generated by limiting dilution. After three rounds of screening and subcloning, stable antibody-producing clones were isolated and adapted to hybridoma-serum-free medium (Life Technologies Corp., Grand Island, NY, USA). Supernatants were collected from selected hybridoma clones and purified through a protein A-sepharose column (GE Healthcare). mAbs were isotyped with the Pierce rapid isotyping kit.

### Binding studies of mAbs using biolayer interferometry

Binding kinetics of MERS-CoV molecules to antibodies were carried out using a FortéBio Octet Red384 instrument. Assays with agitation set to 1,000 r.p.m. in PBS buffer supplemented with 1% BSA (40–50 μl per well) were performed at 30 °C in solid black, tilted-bottom 384-well plates (Geiger Bio-One). Mouse antibodies (40–50 μg ml^−1^) were used to load anti-mouse IgG Fc capture probes for 300 s (s) to capture levels of 1−1.5 nm. Biosensor tips were then equilibrated for 180 s in PBS–1% BSA buffer before binding assessment of MERS-CoV molecules for 300 s followed by dissociation for 300 s. A human-Fc-MERS-CoV S2 chimeric molecule (S2–hFc) was used to load anti-human IgG Fc capture probes for 300 s, and binding to G4 Fab was assessed. Data analysis and curve fitting were carried out with the Octet software, v 8.0.

### X-ray crystallography

A construct encoding the RBD (residues 367–606) with a c-terminal HRV-3c cleavage site and His_6_ purification tag was produced in GnTi^−^ cells. Protein was purified using NiNTA affinity chromatography. The D12 Fab was prepared using the Pierce Mouse IgG1 Fab kit. The RBD molecule was mixed with the D12 Fab in a 1:1.5 molar ratio and was allowed to sit for 30 min at room temperature. All proteins were purified using size exclusion chromatography (Superdex S200) and concentrated to ∼5–8 mg ml^−1^. Crystallization screening was carried out using a Mosquito crystallization robot, using the hanging drop vapour diffusion method at 20 ^o^C by mixing 0.1 μl of protein complex with 0.1 μl of reservoir solution followed by manual optimization.

RBD England1 crystals were obtained using a reservoir solution of 0.1 M Tris-HCl pH 8.5, 10% 2-methyl-2,4-pentanediol (MPD) and 29% polyethylene glycol (PEG) 1,500. Crystal form 1 of the D12 Fab:RBD England1 complex was grown in 0.1 M sodium acetate pH 5.5, 50 mM sodium chloride, 10% PEG 400 and 11% PEG 8,000. Both crystals were cryo-cooled in liquid nitrogen using mother liquor containing 20–22% ethylene glycol as a cryoprotectant. Crystal form 2 of D12 Fab:RBD England1 was grown in 0.1 M sodium Cacodylate pH 6.5, 80 mM magnesium acetate, 14.5% PEG 8,000 using 15% 2R–3R butanediol as a cryoprotectant.

Data were collected at a wavelength of 1.00 Å at SER-CAT beamlines ID-22 and BM-22 (Advanced Photon Source, Argonne National Laboratory). All diffraction data were processed with the HKL2000 suite^46^, structures were solved by molecular replacement using PHASER^47^ and iterative model building and refinement were performed in COOT^48^ and BUSTER-TNT^49^, respectively. For the RBD England1 crystals, a solution was obtained using the PDB ID 4KR0 (ref. 28) molecule B as a search model. For the two crystal forms of the D12: RBD England1 complex, a solution was obtained using PDB ID 4KR0 molecule B as a search model for the RBD, PDB ID 1IGM (ref. 50) as a search model for the Fab variable domain, and the mouse constant region of the Fab F26G19 PDB ID 3BGF^51^ as a search model for the Fab constant domain. In both crystal forms, two RBD–Fab complexes were obtained per asymmetric unit.

A cross-validation test set using 5% of the data was used to assess the model refinement process with structure model validation carried out using MolProbity^52,53^. The RBD England1 model was refined to a final *R*_factor_ value of 22.1% and *R*_free_ value of 25%. The D12: RBD England1 crystal form 1 gave a structure model with a final Rfactor value of 18.5% and Rfree value of 24.8%. The D12: RBD England1 crystal form 2 gave a structure model with a final Rfactor of 22.5% and Rfree value of 26.7%. All structures had 99% residues in the favoured region of the Ramachandran plot.

### MERS-CoV mAb escape mutations

WHO-Vero or Vero81 cells (Vero) were provided by Ralph Baric at the University of North Carolina-Chapel Hill and were maintained in Dulbecco’s modified Eagle’s medium (Invitrogen) containing 7% FBS, supplemented with penicillin, streptomycin and amphotericin B. MERS-CoV EMC strain generated from an infectious clone (from Ralph Baric^54^) was propagated and assessed using plaque assay on Vero cells. All cell and virus incubations were at 37 °C in 5% CO_2_. All viral studies were performed in certified BSL3 laboratories and exclusively within biological safety cabinets using protocols for safe study, maintenance and transfer that have been reviewed and approved by the Institutional Biosafety Committees of the Vanderbilt University.

### Plaque reduction neutralization assay

Starting at a concentration of 10 μg ml^−1^, mAbs were serially diluted fivefold and mixed with an equal amount of virus for a total of six times. Virus–mAb mixtures were incubated at 37 ^o^C for 30 min, and then 200 μl of each mixture was used to inoculate Vero cell monolayers in six-well plates in duplicate. Following 1-h incubation, cells were overlaid with complete media plus 1% agar. Plaques were visualized and counted between 48 and 52 h post infection. Amount of infectious virus in the presence of each mAb concentration was calculated and graphed.

### Passage for antibody escape

mAb was added to each 25-cm^2^ flask of Vero cells in 3 ml DMEM containing 3.5% serum, followed by MERS-CoV at an multiplicity of infection of 0.1 p.f.u. ml^−1^. Two days later, mAb and 10 μl of the supernatant from the previous infection was added to new flasks of Vero cells. Three separate lineages were carried in parallel, with increasing amounts of mAb at each passage. Following the fifth passage, the supernatant was removed, aliquoted and frozen, and then thawed and titred using plaque assay in the presence and absence of mAb. Ten plaques from wells with mAb from each lineage were picked, and then used to inoculate a 25-cm^2^ flask for 2 days. The supernatant was removed, aliquoted and frozen. Cells were lysed using TRIzol reagent (Life Technologies Corp.) according to the manufacturer’s instructions and frozen.

### Sanger (dideoxy) sequence analysis of the Spike glycoprotein

Total cellular RNA was extracted from lysates infected with mAb-resistant plaque clones and then subjected to RT–PCR, and the MERS-CoV Spike gene was amplified in two overlapping amplicons using primers 5′- CCTCATCTGAAGGATTCC -3′; 5′- AATCATTGTCCTGCTGGC -3′; 5′- CACCAATAGTGTTTGCCC -3′; 5′- CATACCAGGTTTTGGAGG -3′; 5′- GATGGTCCTACCGAACAT -3′; 5′- TATACGCAGAGCTATAGACT -3′. Each amplicon was sequenced to give complete gene coverage. The resulting sequences were assembled and compared with the expected, theoretical sequence for MERS-CoV Spike.

### Statistical analysis

We calculated GMT and 95% confidence intervals for all antibody titres. The means and s.e.’s were calculated for all other data. *P* values were calculated with a two-tailed, unpaired, nonparametric Mann–Whitney test using the Prism software (Version 6.04, GraphPad, La Jolla, CA). Statistically significant differences met a threshold (*α*) of 0.05. Statistical variation within each data set is represented as the standard error in each of the figures. Pearson correlation coefficients and associated *P* values were calculated using the Prism software. Variances were generally similar to justify the use of nonparametric statistical tests.

## Additional information

**How to cite this article:** Wang, L. *et al*. Evaluation of candidate vaccine approaches for MERS-CoV. *Nat. Commun.* 6:7712 doi: 10.1038/ncomms8712 (2015).

## Supplementary information

Supplementary Information Supplementary Figures 1-15, Supplementary Tables 1-2 and Supplementary References (PDF 4671 kb)

## Data Availability

Protein Data Bank
4ZPT
4ZPV
4ZPW
