# Supplementary material for: Evaluation of candidate vaccine approaches for MERS-CoV
Source: Nat Commun. 2015 Jul 28;6:7712. doi: 10.1038/ncomms8712 (PMC4525294; doi:10.1038/ncomms8712)
Supplement: Supplementary Information — Supplementary Figures 1-15, Supplementary Tables 1-2 and Supplementary References (PDF 4671 kb) [file 41467_2015_BFncomms8712_MOESM309_ESM.pdf]

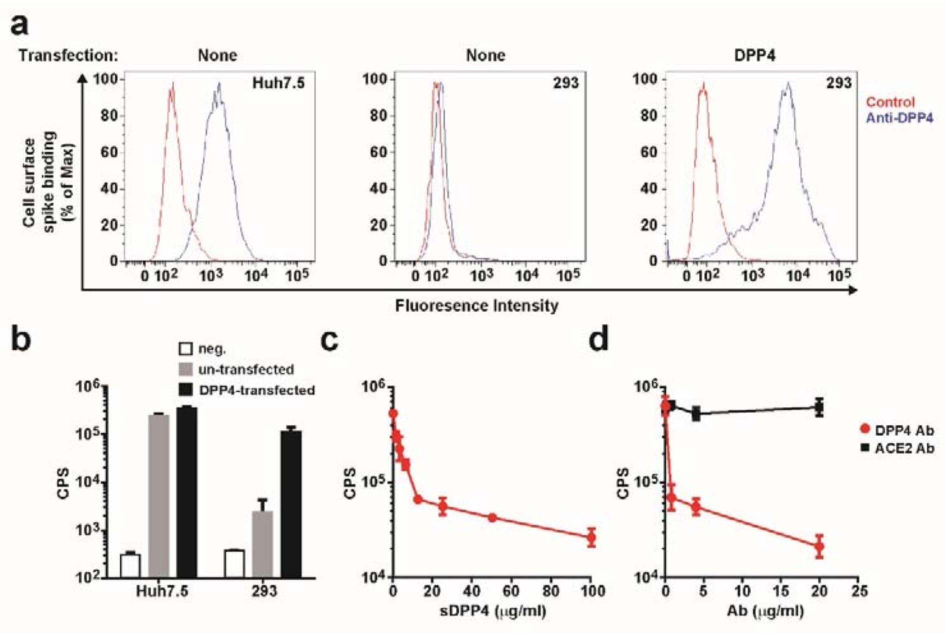

Supplementary Figure 1. MERS-CoV pseudovirus utilizes human DPP4 to transduce target cells. (a) DPP4 expression on the cell surface. Huh7.5 cells (left panel), DPP4-untransfected HEK 293 cells (middle panel), and DPP4-transfected HEK 293 cells (right panel) were stained with goat anti-DPP4 antibody (in blue) and control antibody (in red) and analyzed by flow cytometry. **(b)** Transduction of DPP4-expressing cells by pseudotyped MERS-CoV England1 virus. Huh7.5 and HEK 293 cells without and with DPP4-transfection (in grey and black bars) were transduced by MERS-CoV pseudotyped virus. Relative expression of luciferase activity was measured (CPS). Untransfected and untransduced cells were used as the background control (open bars). Each bar represents the mean of triplicate assays with standard errors. One representative of two experiments is shown. **(c)** Transduction of Huh7.5 cells by MERS-CoV pseudovirus was blocked by soluble human DPP4 (sDPP4). MERS-CoV England1 pseudovirus was incubated with soluble human DPP4 before transduction of Huh7.5 cells. Relative luciferase activity (CPS) is shown. **(d)** Transduction of Huh7.5 cells by MERS-CoV pseudovirus was blocked by anti-DPP4, but not anti-ACE2 antibody. Huh7.5 cells were incubated with anti-DPP4 or anti-ACE2 polyclonal antibodies and then transduced with MERS-CoV England1 pseudovirus. Relative luciferase activity (CPS) is shown. Each data point in (c) and (d) represents the mean of triplicate assays with standard errors. One representative of two repeated experiments is shown.

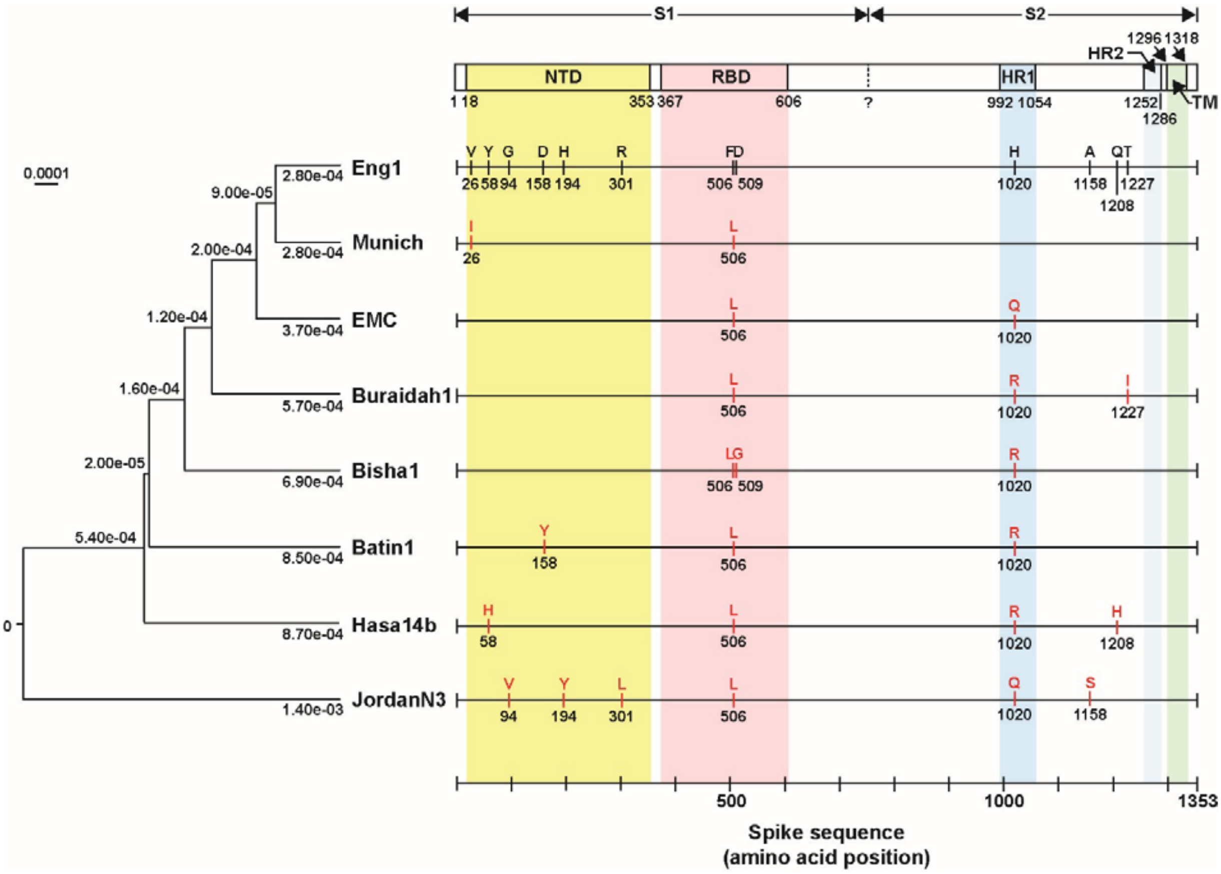

Supplementary Figure 2. Comparison of MERS-CoV Spike (S) glycoprotein sequences across strains used for a pseudotyped virus neutralization assay panel. A schematic representation of MERS-CoV S protein is shown with the N-terminal domain (NTD) highlighted in yellow, receptor binding domain (RBD) in pink, heptad repeats 1 and 2 (HR1 and HR2) in blue, and transmembrane domain (TM) in green. Eight MERS-CoV S sequences published in GenBank were aligned with the England1 strain. The amino acid differences are shown in red with the England1 strain as the referent. Phylogenetic distance between strains is represented by branch length on the phylogenetic tree to the left of the sequences.

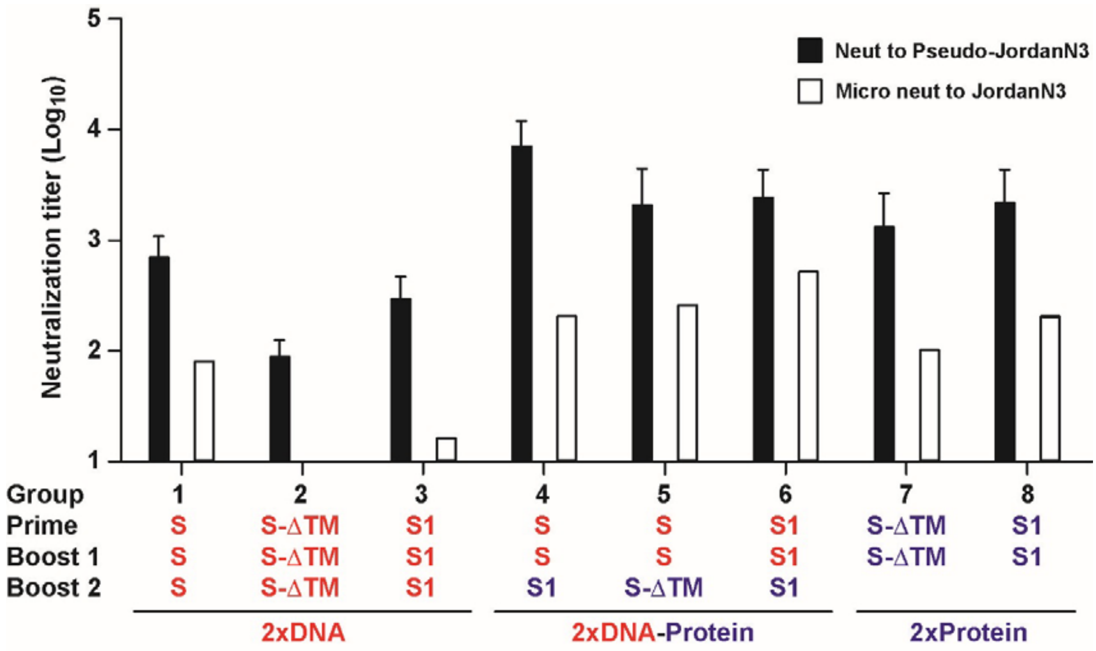

Supplementary Figure 3. MERS-CoV vaccine elicited virus neutralization responses as measured by both pseudotyped and live virus neutralization assays. Eight groups of mice (fiver per group) were immunized as indicated in Figure 1. Neutralizing antibodies from sera five weeks after last vaccine boost were measured by a pseudovirus neutralization assay (black bars) and live virus micro-neutralization assay (open bars) to MERS-CoV JordanN3 respectively. Each black bar represents the mean of triplicate assays with standard errors. Each open bar represents a single assay.

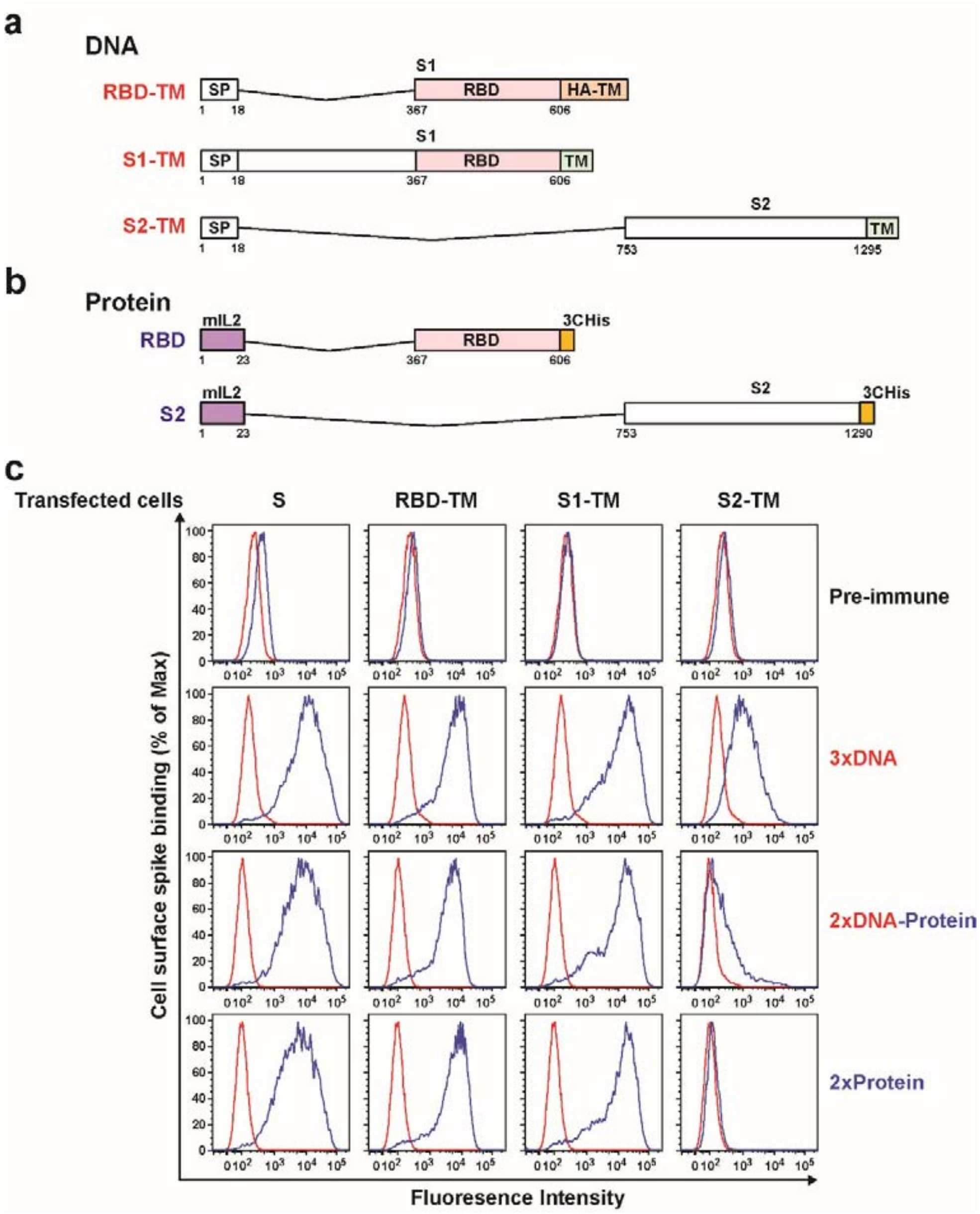

Supplementary Figure 4. MERS-CoV S DNA immunization induced antibody binding to both S1 and S2. (a, b) Schematic representation of MERS-CoV Spike DNA and protein constructs used for cell adsorption assays. **(c)** Sera from mice immunized with MERS-CoV S DNA, primed with S DNA and boosted with S1 protein plus Ribi adjuvant, or primed and boosted with S1 protein plus Ribi adjuvant were assayed by flow cytometry for their binding to cell surface-expressed MERS-CoV Spike proteins. HEK 293T cells transfected with MERS-CoV S, RBD-HATM, S1-TM and S2-TM were incubated with sera from the three immunization groups (1:200 dilution) and then stained with anti-mouse PE conjugate. One representative of three repeated experiments is shown. Red and blue lines indicate untransfected or anchored-S, RBD-TM, S1-TM and S2-TM transfected cells (as labeled on the top of the figure) stained with the sera labeled at the right. S: Spike glycoprotein, RBD: receptor binding domain, HA: hemagglutinin, TM: transmembrane.

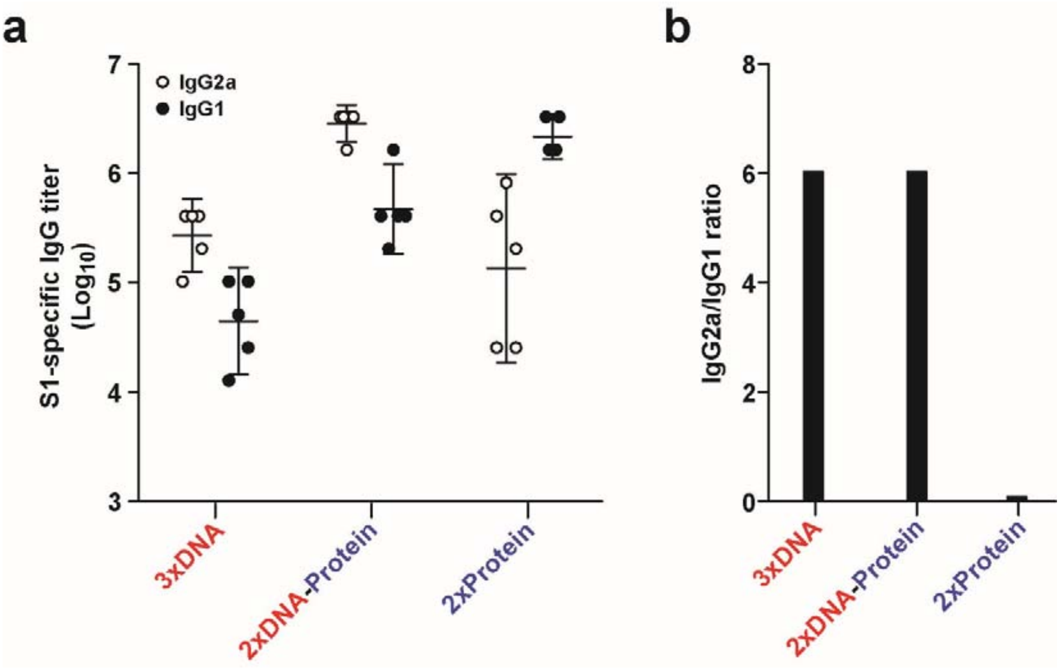

Supplementary Figure 5. MERS-CoV S DNA/S1 protein prime-boost vaccination induced a Th1-biased IgG response compared to a Th2-biased response elicited by a S1 protein prime-boost regimen. (**a**) Sera from mice (5 per group) immunized with MERS-CoV S DNA, primed with S DNA and boosted with S1 protein plus Ribi adjuvant, or primed and boosted with S1 protein plus Ribi adjuvant were assayed, by ELISA, for their predominance of MERS-CoV S1-specific IgG1 and IgG2a antibody responses. Open and black circles represent IgG2a and IgG1 antibody titers (Geometric mean titer (GMT) with 95%CI), respectively. One of two repeated experiments is shown. (**b**) The GMT ratios of IgG2a to IgG1 in the three groups were calculated from the left panel.

	mAb	Neutralization	Binding			
			ELISA			Western Blot
		Pseudovirus (England1)	RBDs	S1	S-ΔTM	Denatured S
RBD specific	A8	83.7	3.04	2.96	0.57	No
	C7	83.7	3.11	1.95	0.27	No
	C8	99.7	3.03	0.59	0.14	No
	C9	95.6	3.11	0.77	0.12	No
	C10	77.1	3.23	0.69	0.10	No
	C12	88.3	3.19	0.73	0.05	No
	D2	99.0	3.23	3.32	0.22	No
	D6	44.5	3.14	3.01	0.28	No
	D10	72.3	3.14	3.24	0.07	No
	D12	99.8	3.31	3.28	0.05	No
	E2	77.1	2.96	0.95	0.05	No
	F10	99.2	3.17	2.64	0.11	No
	F11	99.4	3.16	2.67	0.09	No
S1 specific (non-RBD)	A6	70.0	0.06	1.88	2.75	No
	A7	77.8	0.09	2.74	1.18	No
	B4	32.2	0.05	0.87	0.05	No
	B5	79.0	0.04	2.80	0.14	No
	B11	65.4	0.05	0.74	0.96	No
	B12	60.4	0.06	1.27	0.10	No
	C1	21.6	0.03	2.82	0.14	Yes
	C2	20.0	0.04	2.94	1.16	Yes
	C3	-1.7	0.05	2.97	0.34	No
	D1	23.5	0.06	3.32	0.55	Yes
	D8	8.08	0.04	1.85	0.12	No
	F1	64.3	0.03	3.03	0.32	No
	F4	2.2	0.05	3.17	2.35	No
	F6	7.2	0.05	2.08	0.36	No
	G2	80.9	0.04	3.15	0.58	No
	G3	23.0	0.05	2.70	0.27	No
	H5	4.8	0.05	3.09	2.81	No
	H6	3.4	0.05	3.03	2.75	No
	H7	-2.0	0.04	3.05	2.76	No
S2 specific	A3	85.3	0.25	0.05	2.33	Yes
	A4	1.4	0.04	0.04	2.24	No
	A10	85.3	0.05	0.05	2.48	Yes
	A12	79.9	0.06	0.05	1.32	No
	B2	39.2	0.04	0.05	2.12	No
	C6	83.6	0.04	0.05	3.07	No
	D5	50.7	0.04	0.05	2.69	Yes
	F7	28.5	0.06	0.10	3.12	Yes
	F8	59.9	0.06	0.06	3.01	Yes
	F9	61.6	0.10	0.10	1.56	No
	G1	69.1	0.06	0.05	3.09	Yes
	F3	94.3	0.05	0.05	2.45	No
	G4	87.5	0.05	0.06	2.80	No

Supplementary Figure 6. Identification of monoclonal antibodies against the MERS-CoV Spike glycoprotein. Immunized mice in both DNA and protein vaccine groups had their spleens harvested three days after an additional S1 protein. Splenocytes were then fused with Sp2/0 myeloma cells to generate hybridomas that underwent three rounds of screening for binding to the S1, RBD, and S2 domains. The final round of screens generated 45 subclones. Supernatant from the subclones culture were subjected to neutralization and binding tests. Percentage of neutralization against pseudoviruses of Eng1 strain from the subclones was determined and is shown highlighted from high (red > 90%) to low (green < 1%). Supernatants from the subclones were assessed for binding to the RBDs, S1 and S-ΔTM proteins. OD450nm values are indicated for strong binding (red, > 3) to weak binding (green, < 1). Western blot analysis was done to assess whether the subclones can recognize denatured S linear eptitopes. Based on the ELISA binding data, the mAbs were classified into three groups: RBD specific, S1 specific (non-RBD) and S2 specific as indicated. Four of these mAbs (D12, F11, G2 and G4) were selected for additional characterization based on their antigenic specificity and high neutralization potency.

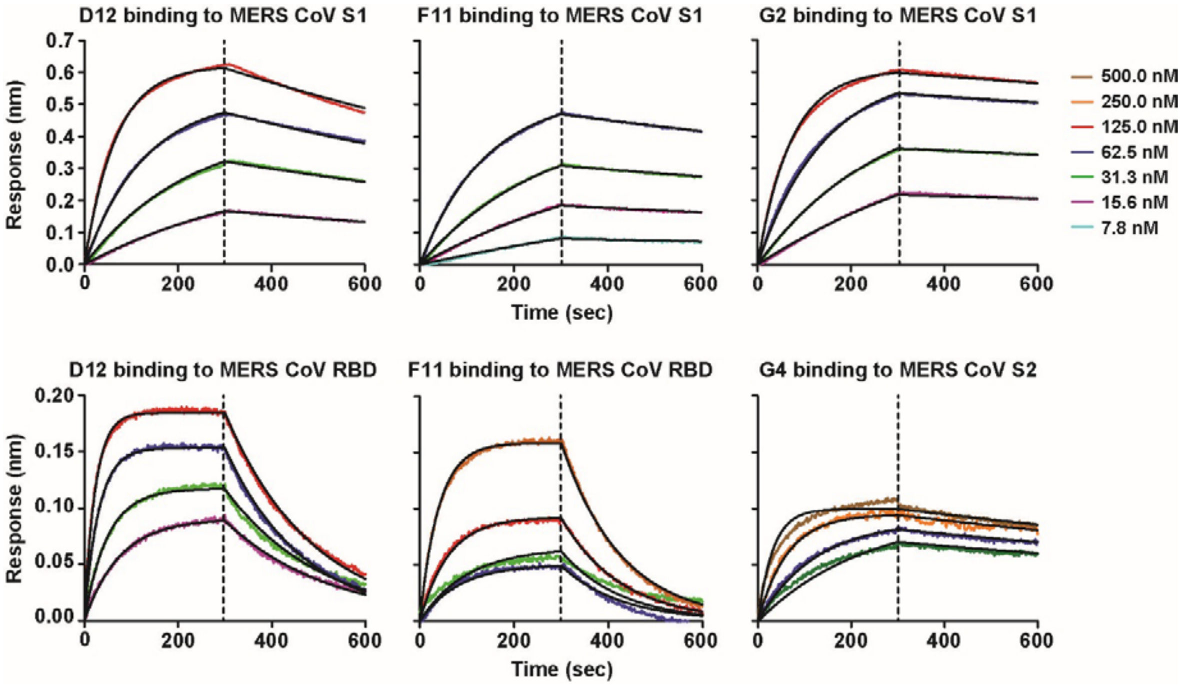

Supplementary Figure 7. Octet Biosensorgrams of MERS-CoV S1, RBD, S2 molecules binding to vaccine-induced mouse monoclonal IgGs. Mouse monoclonal antibodies were loaded onto AMC probes and association with MERS-CoV antigen was allowed to proceed for 300 s, followed by dissociation for 300 s with the responses measured in nm using an Octet Red 384 machine. The S2 binding to G4 was measured by loading human-Fc-S2 onto AHC probes and measuring association with varying concentrations of G4 Fab. The solid black lines represent the best fit of the kinetic data to a 1:1 binding model. All experiments were carried out, in triplicate, at 30°C in PBS buffer (pH 7.4) supplemented with 1% BSA to minimize non-specific binding. The dotted line indicates the beginning of dissociation and the legend indicates the MERS CoV antigen and G4 Fab concentrations used.

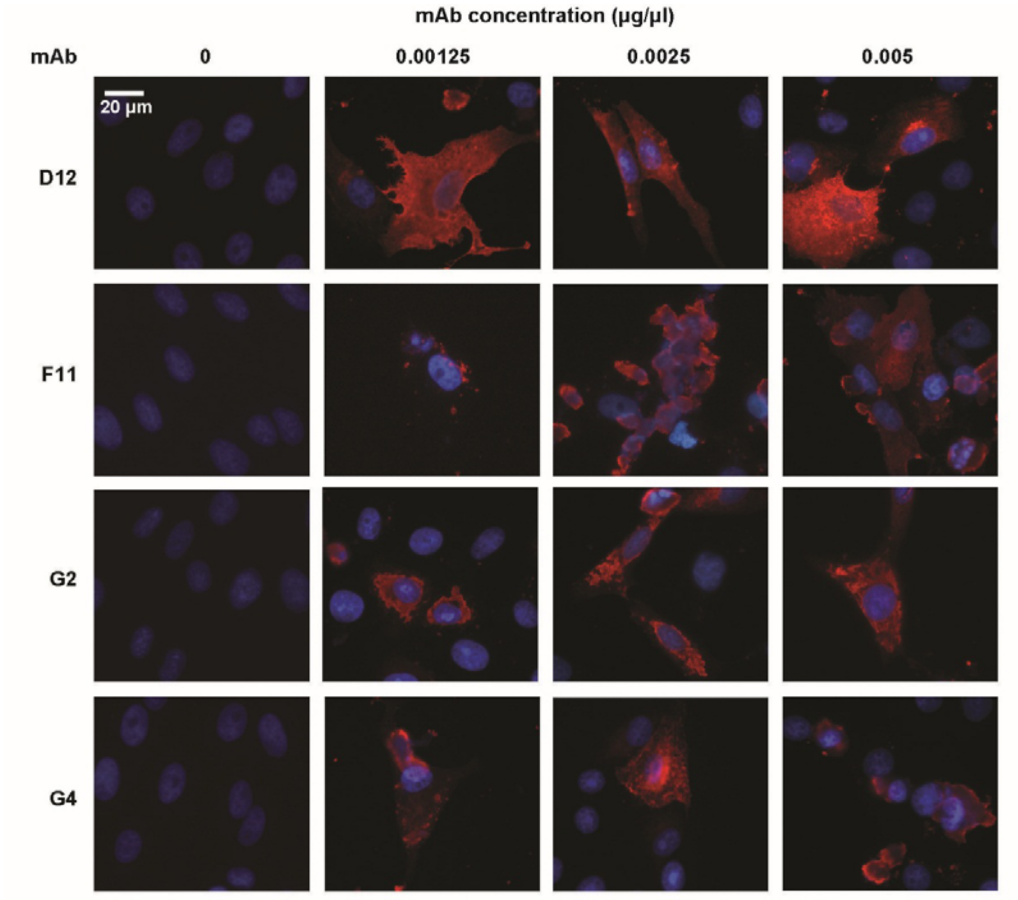

Supplementary Figure 8. Immunoflourescence of monoclonal antibodies (mAb) specific for MERS-CoV infected cells. Red Alexa Fluors 546 signal for isolated mAbs D12, F11, G2 and G4 binding to MERS-CoV (EMC strain)-infected Vero cells was robust for serial mAb dilutions down to 0.00125 μg/μL. Blue regions indicated the nucleus of the Vero cells and 0 μg/ml mAb concentration was used as the control.

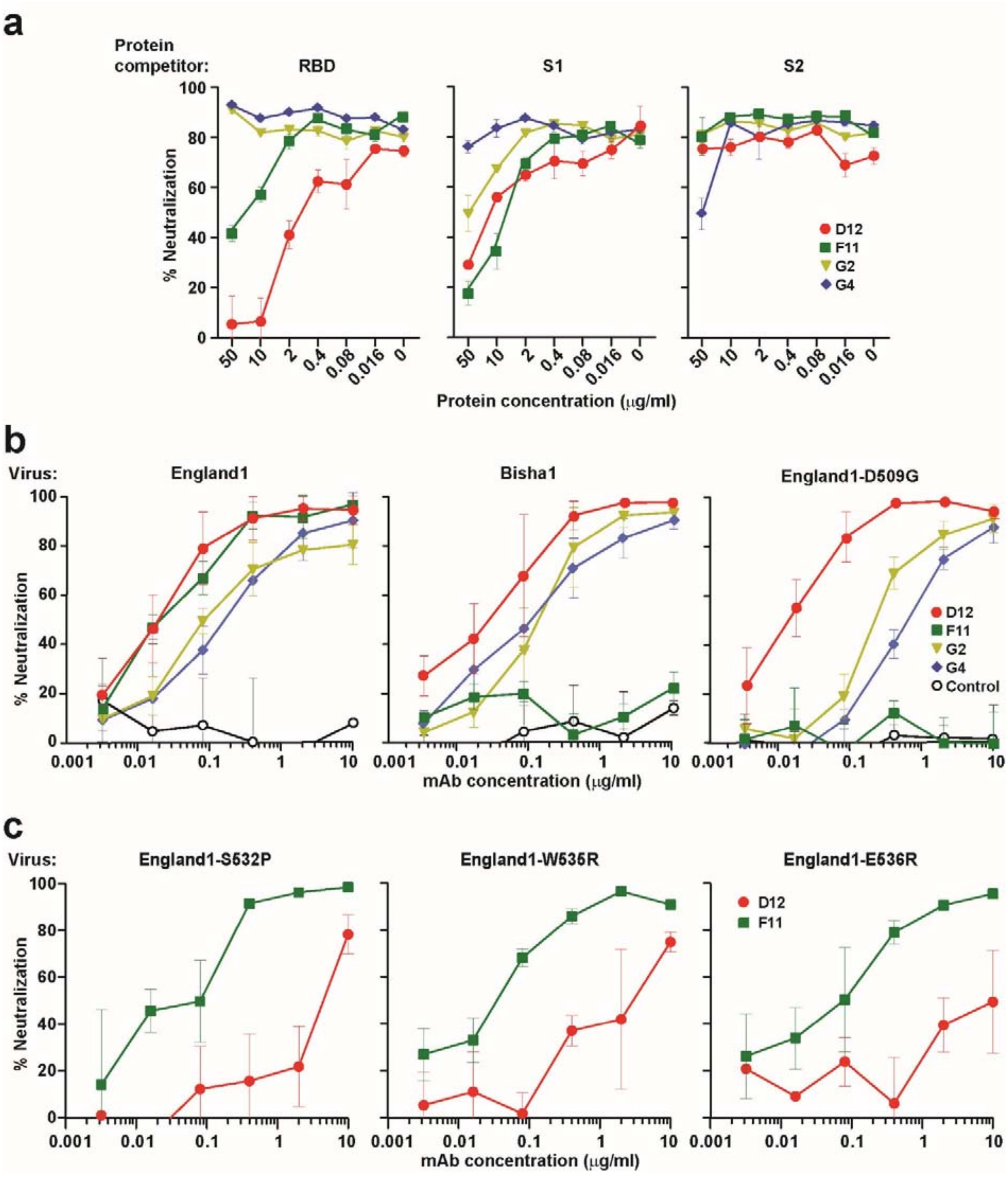

Supplementary Figure 9. MERS-CoV Spike glycoprotein monoclonal antibody (mAb) antigenic specificity. (**a**) Protein competition neutralization assay. mAbs at a single dilution were assayed for neutralization of MERS-CoV England1 pseudovirus in the presence of soluble MERS-CoV RBD, S1 and S2 proteins at concentrations of 0.016 to 50 μg/ml. (**b, c**) mAbs were assayed for neutralization to the MERS-CoV or mutant pseudotyped viruses. Data are presented as the mean of triplicates with standard errors. One representative of two repeated experiments is shown.

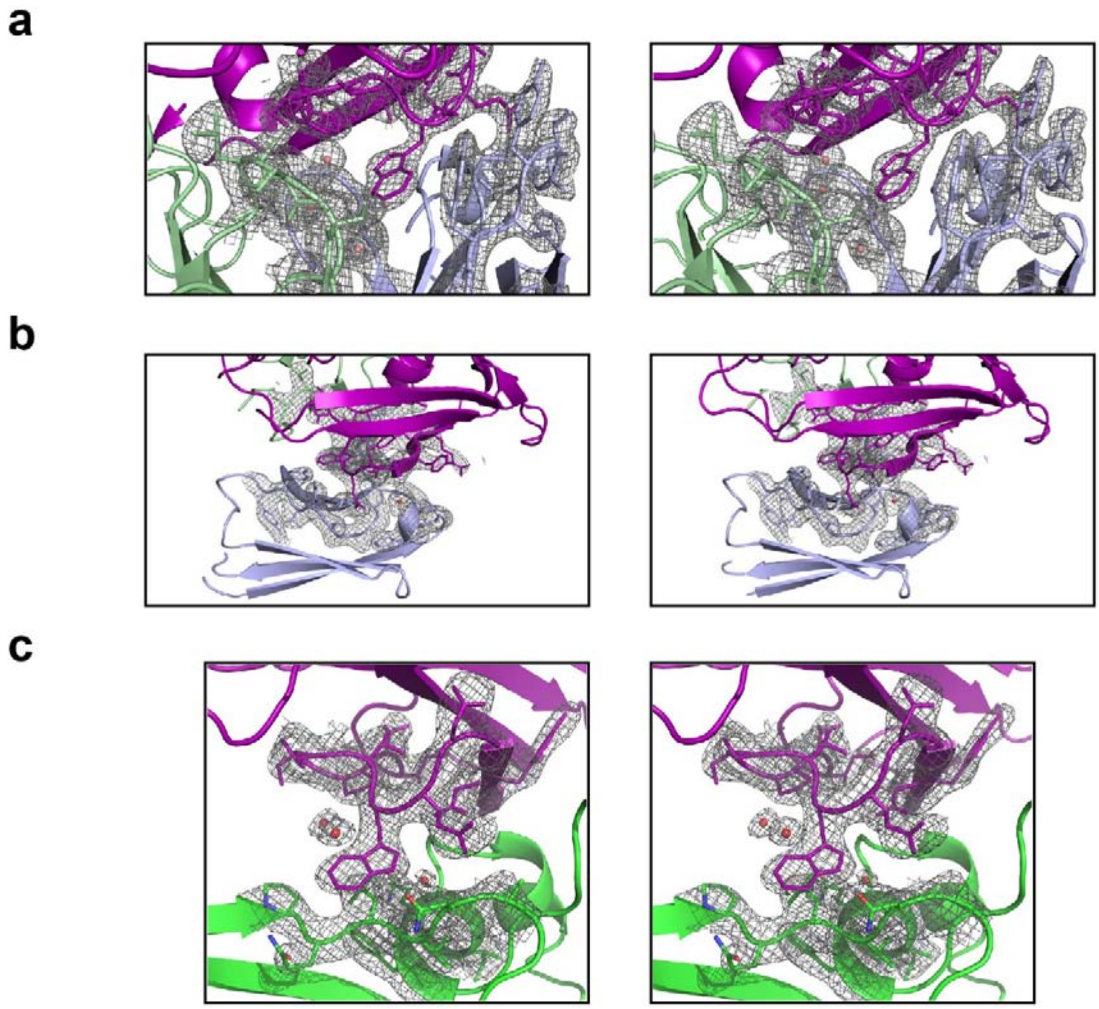

Supplementary Figure 10. MERS-CoV RBD and antibody crystal structure electron density stereo images. (a) Electron density stereo image ($2F_o$-F_c map at 1 σ contour level) of the MERS-CoV RBD in complex with neutralizing antibody D12 crystal form 1 at 2.65 Å resolution. The image is oriented as in Fig. 4c with the MERS RBD W535 located in the center, the heavy chain is colored blue and the light chain is colored green. **(b)** Electron density stereo image ($2F_o$-F_c maps at 1 σ contour level) of the MERS-CoV RBD in complex with neutralizing antibody D12 crystal form 2 at 3.25 Å resolution. The structure is rotated to allow visualization of E536 interacting with the D12 CDR H2 loop. **(c)** Electron density stereo image ($2F_o$-F_c maps at 1 σ contour level) of the MERS-CoV RBD *England1* at 3.17 Å resolution. Two MERS RBD molecules are located within the asymmetric unit and the W535 interacts with the neighboring molecule.

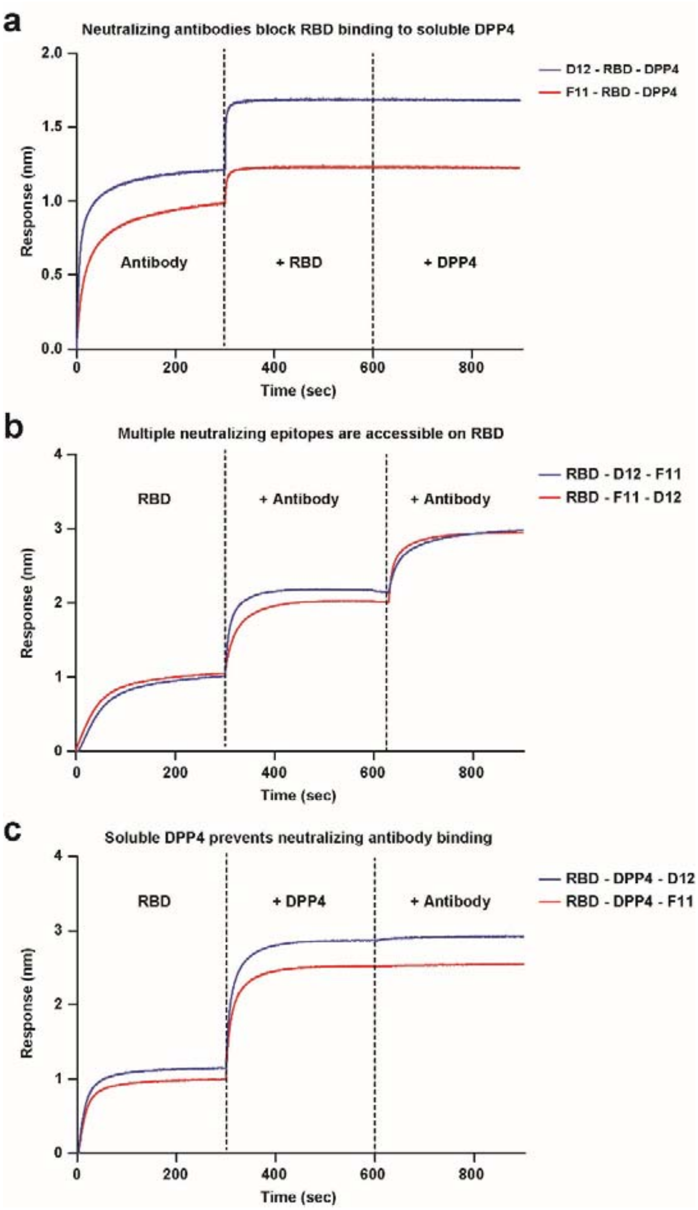

Supplementary Figure 11. Assessment of D12 and F11 interactions with MERS CoV RBD. (**a**) D12 and F11 directly block RBD binding to DPP4. Mouse monoclonal antibodies D12 and F11 were loaded onto AMC probes for 300 s and association with MERS-CoV RBD was allowed to proceed for 300 s, followed by incubation with soluble DPP4 for 300 s with the responses measured in nm using an Octet Red 384 machine. (**b**) Multiple neutralizing epitopes are accessible on MERS CoV RBD. MERS CoV RBD was loaded onto anti-penta-His probes for 300 s followed by sequential binding of both D12 and F11 mAbs. (**c**) Soluble DPP4 prevents binding of RBD to D12 and F11 mAbs. MERS CoV RBD was loaded onto anti-penta-His probes for 300 s followed by sequential binding of DPP4 and either D12 or F11 mAbs. All experiments were carried out at 30°C in PBS buffer (pH 7.4) supplemented with 1% BSA to minimize non-specific binding. The dotted line indicates the beginning of incubation with the second and third ligand in each of the experiments.

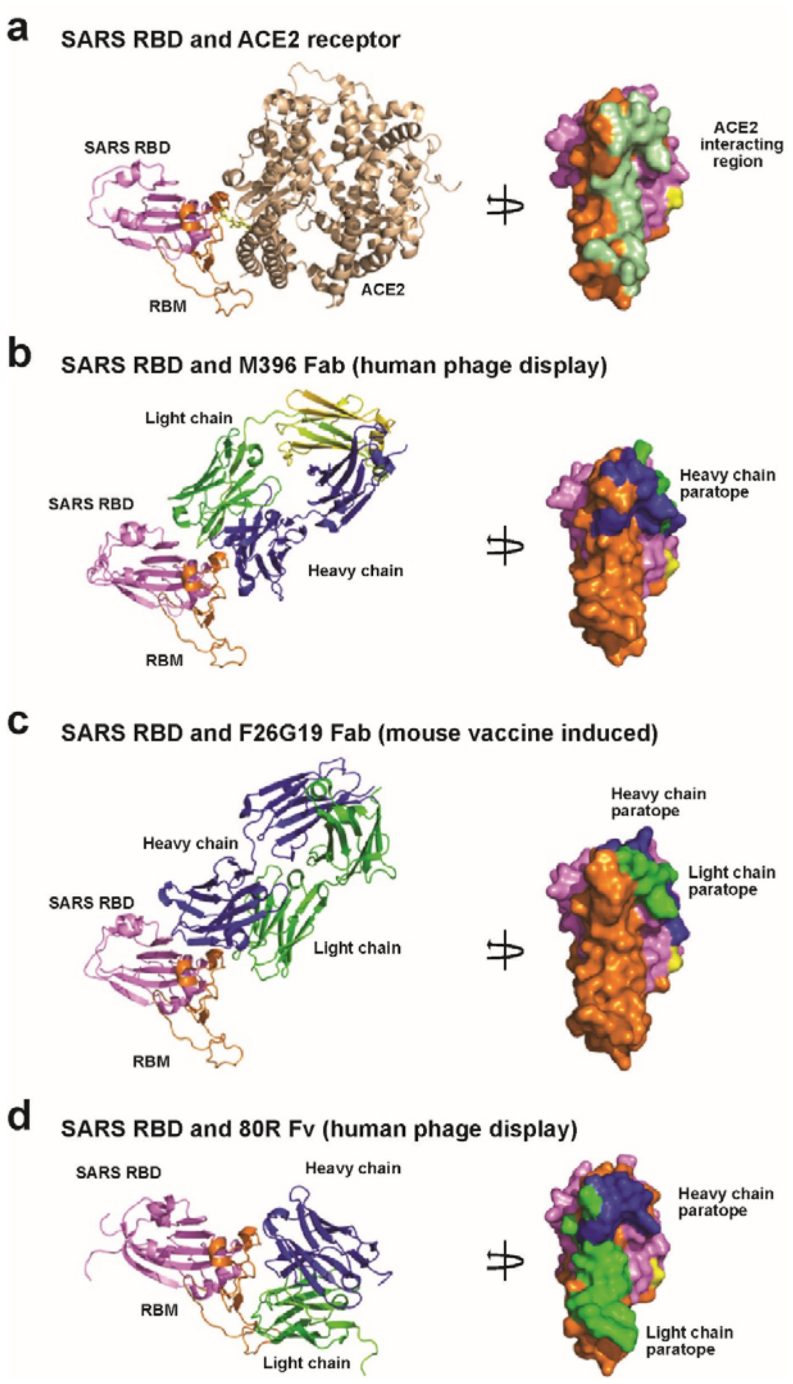

Supplementary Figure 12. Published structures of SARS-CoV neutralizing antibodies that effectively block the receptor interacting region of the virus.
(**a**) SARS receptor binding domain (RBD) (violet) with the receptor binding motif (RBM) (orange) and the ACE2 receptor (green) are shown in cartoon representation. The ACE2 interacting region is mapped onto the SARS RBD (surface representation, rotated) and colored in green. The RBD region that interacts with the ACE2 glycan is shown in yellow. (**b-d**) SARS RBD and M396 Fab[1,2], F26G19 Fab[3] and 80R Fv[4] are shown in cartoon representation. The antibody interacting region is mapped onto the RBD and colored green (Light chain) and blue (Heavy chain).

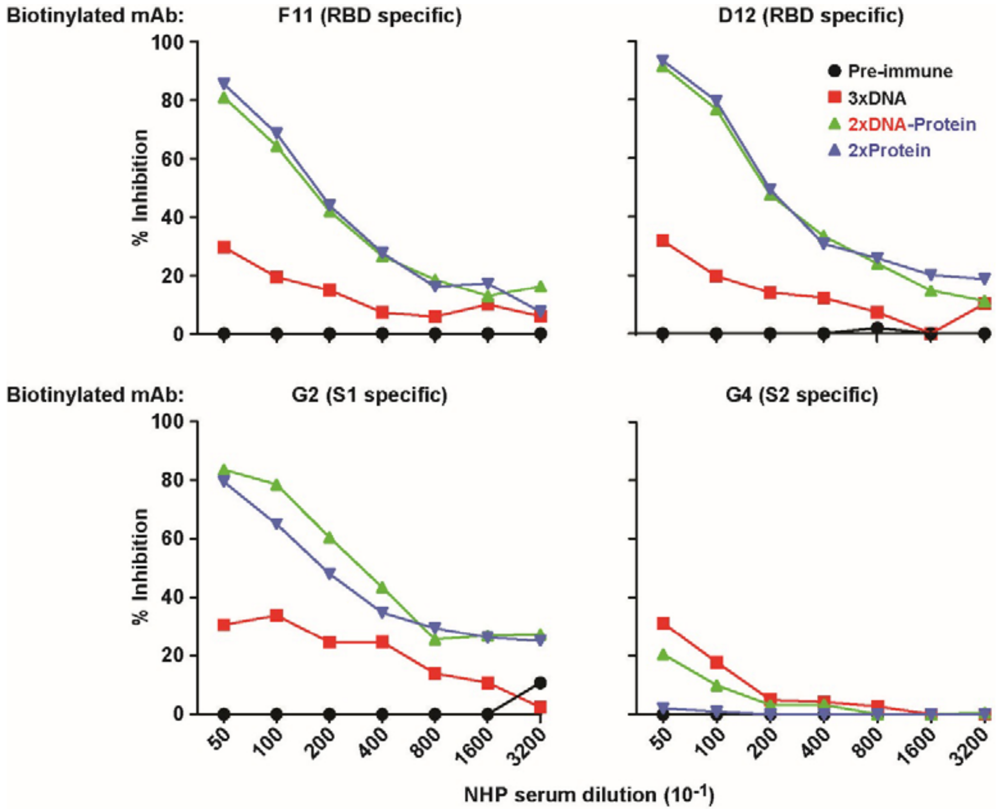

Supplementary Figure 13. Sera from vaccinated non-human primates (NHPs) blocked the binding of murine monoclonal antibodies to MERS-CoV Spike protein. Serial dilutions of mixed NHP sera from three vaccinated groups were tested in competition with biotinylated monoclonal antibodies F11, D12, G2, G4 for binding to MERS-CoV S1 or S-dTM (for mAb G4). Percent inhibition is shown.

a

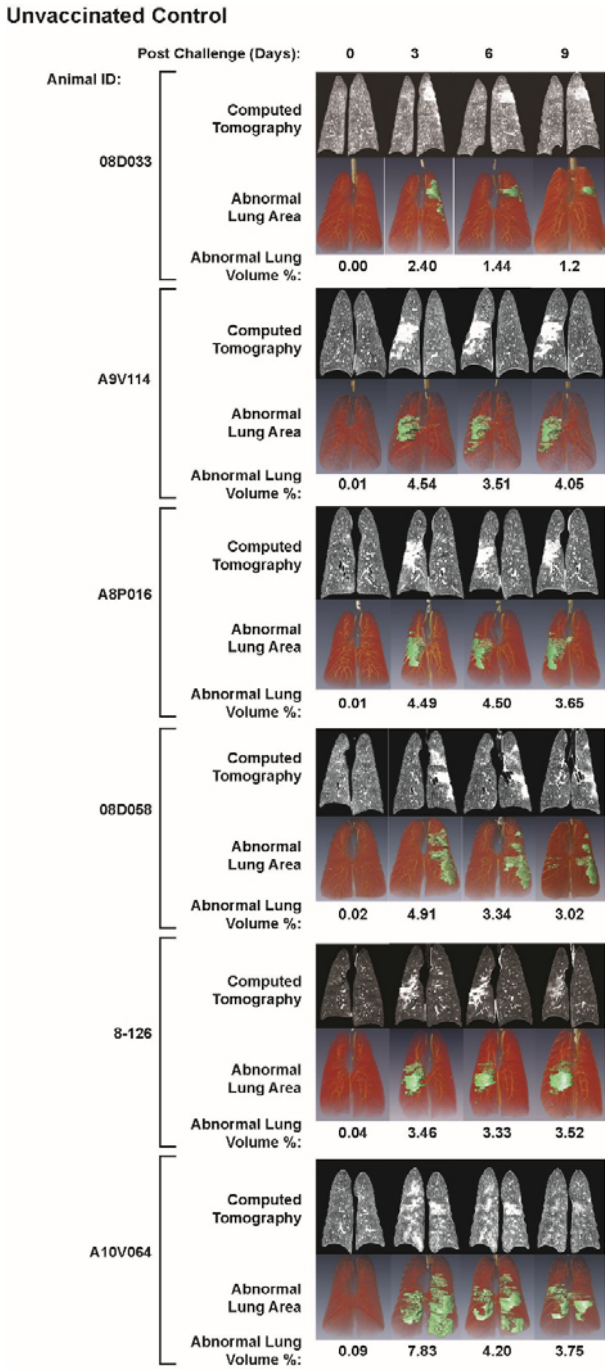

Supplementary Figure 14a. Three dimensional computed tomography (CT) visualizations of lungs from non-human primates (NHPs) challenged with MERS-CoV. (**a-c**) Unvaccinated NHPs (**a**) and those vaccinated with S DNA/S1 protein (**b**) or S1 protein/S1 protein (**c**) underwent chest CT imaging before virus challenge and days 3, 6, 9, and 14 (not shown) post-challenge. Two dimensional coronal CT images and three dimensional reconstructions showed larger volumes of percent abnormal lung (infiltrate, consolidation, ground glass opacity) in the unvaccinated compared to vaccinated NHPs.

b

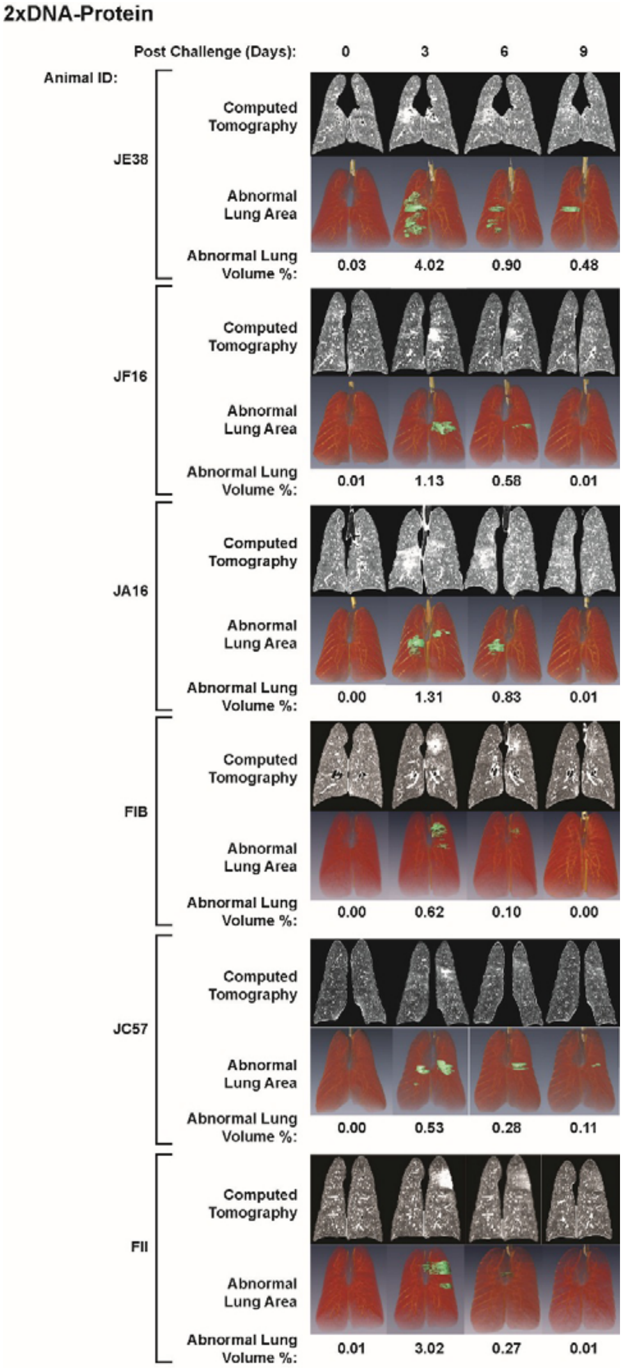

Supplementary Figure 14b. Three dimensional computed tomography (CT) visualizations of lungs from non-human primates (NHPs) challenged with MERS-CoV. (**a-c**) Unvaccinated NHPs (**a**) and those vaccinated with S DNA/S1 protein (**b**) or S1 protein/S1 protein (**c**) underwent chest CT imaging before virus challenge and days 3, 6, 9, and 14 (not shown) post-challenge. Two dimensional coronal CT images and three dimensional reconstructions showed larger volumes of percent abnormal lung (infiltrate, consolidation, ground glass opacity) in the unvaccinated compared to vaccinated NHPs.

c

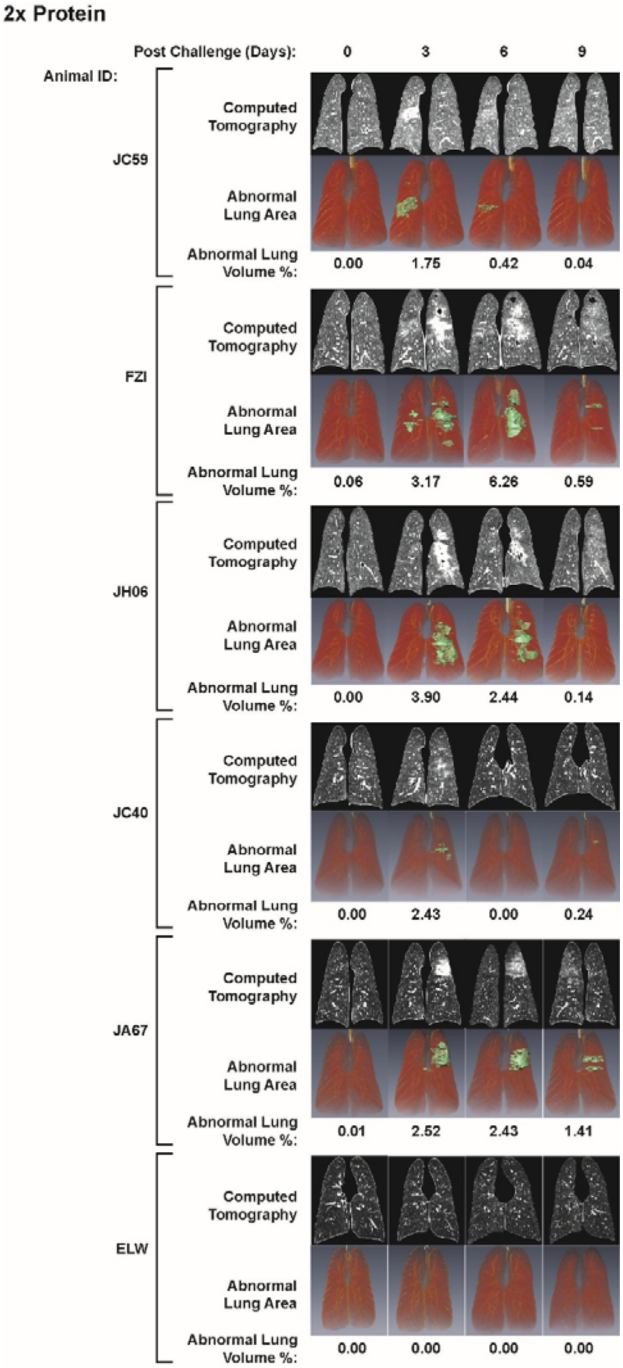

Supplementary Figure 14c. Three dimensional computed tomography (CT) visualizations of lungs from non-human primates (NHPs) challenged with MERS-CoV. (**a-c**) Unvaccinated NHPs (**a**) and those vaccinated with S DNA/S1 protein (**b**) or S1 protein/S1 protein (**c**) underwent chest CT imaging before virus challenge and days 3, 6, 9, and 14 (not shown) post-challenge. Two dimensional coronal CT images and three dimensional reconstructions showed larger volumes of percent abnormal lung (infiltrate, consolidation, ground glass opacity) in the unvaccinated compared to vaccinated NHPs.

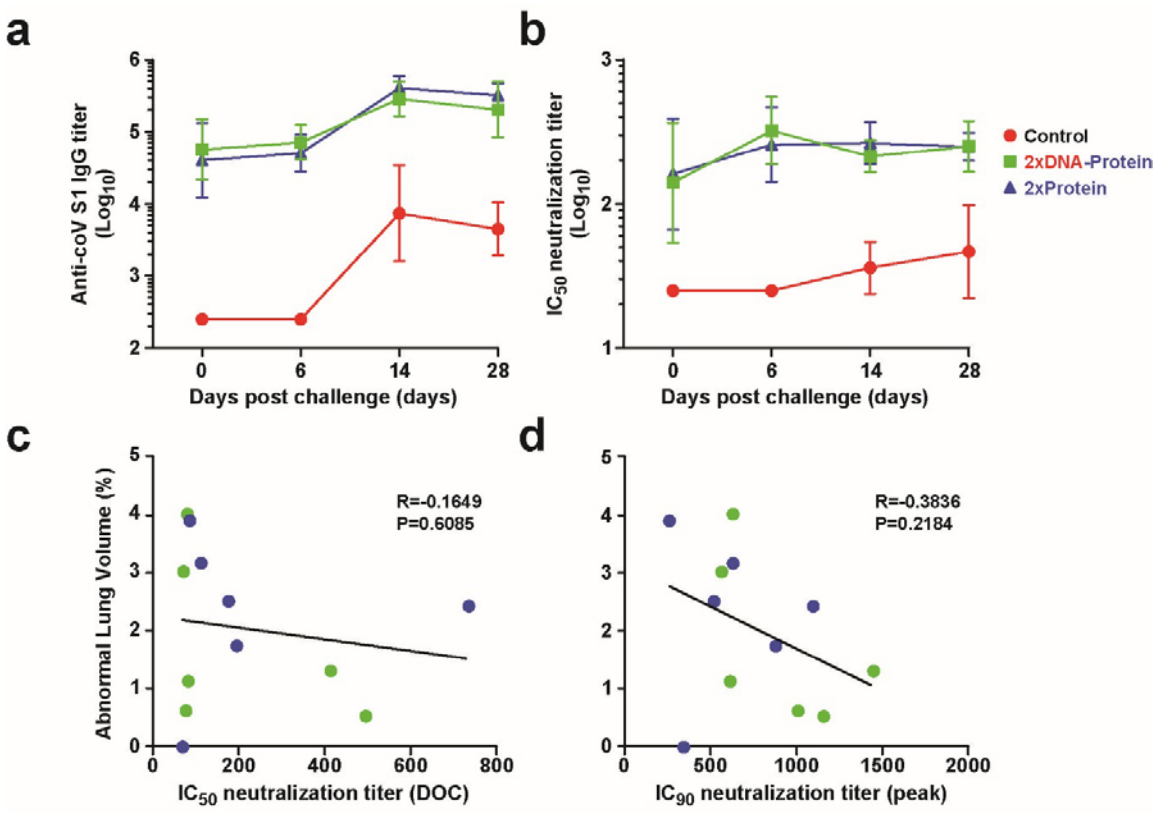

Supplementary Figure 15. Non-human primate (NHP) anti-MERS-CoV antibody responses increase post-challenge but do not correlate with pulmonary disease. (a) ELISA IgG antibody titers and **(b)** neutralization titers both rise after challenge with MERS-CoV. There was no significant correlation between neutralization titers of NHP sera at day of challenge **(c)** or at peak (2 weeks after last boost) **(d)** and lung disease as measured by percent abnormal lung volume on computed tomography. Graphpad Prism 6 was used to determine the Pearson correlation coefficients and corresponding p values.

Supplementary Table 1. Crystallographic data collection and refinement statistics

	RBD England1 + D12 crystal form 1	RBD England1 + D12 crystal form 2	RBD England1
**Data collection**			
Growth condition	0.1 M sodium acetate pH 5.5, 50 mM sodium chloride, 10 % PEG 400, 11 % PEG 8,000	0.1 M sodium cacodylate pH 6.5, 80 mM magnesium acetate, 14.5 % PEG 8,000	0.1 M Tris-HCl pH 8.5, 10 % MPD, 29 % PEG 1,500
Space group	$P2_12_12_1$	$P2_12_12_1$	$P2_12_12_1$
Cell constants			
a, *b*, *c* (Å)	74.5, 128.8, 170.9	76.2, 106.1, 171.1	46.7, 109.9, 125.3
α, β, γ (°)	90.0, 90.0, 90.0	90.0, 90.0, 90.0	90.0, 90.0, 90.0
Resolution (Å)	50.0-2.65 (2.71-2.65)	50.0-3.25 (3.36-3.25)	50.0-3.17 (3.31-3.17)
R_{merge}	11.5	13.3	17.8
No. unique reflections	75350 (3346)	40088 (2566)	10616 (903)
$I / \sigma I$	9.3 (2.0)	32.4 (2.0)	5.7 (2.0)
Completeness (%)	999.2 (99.7)	85.4 (76.3)	91.7 (81.3)
Redundancy	4.5 (3.7)	5.2 (2.1)	4.8 (2.3)
**Refinement**			
Resolution (Å)	48.70-2.65 (2.72-2.65)	50.0-3.25 (3.33-3.25)	43.7-3.17 (3.31-3.17)
No. Reflections			
R_{work} / R_{free} (%)	18.5/24.8	22.5/26.7	22.1/25.0
No. atoms			
Protein	9710	9652	3222
Ligand/ion	762	-	167
Water	734	56	106
B-factors			
Protein	40.0	77.0	60.25
NAG/ion	110	-	127.2
Water	46.0	82.4	44.1
R.m.s. deviations			
Bond lengths (Å)	0.010	0.006	0.010
Bond angles (°)	1.2	1.42	1.22

Each crystal structure was determined using a single crystal.
Values in parentheses are for the highest-resolution data shell.

Supplementary Table 2a. D12 antibody interactions with MERS England1 RBD.

	D12 antibody	MERS RBD	Distance (Å)
Hydrogen Bonds			
	H:TYR 32[OH]	S:ARG 542[NE]	3.1
	H:TYR 32[OH]	S:ARG 542[NH2]	3.3
	H:SER 52[OG]	S:GLU 536[OE1]	2.5
	H:SER 52a[OG]	S:ASP 539[OD1]	2.6
	H:GLY 53[N]	S:GLU 536[OE1]	3.3
	H:THR 55[N]	S:GLU 536[OE1]	3.6
	H:THR 55[OG1]	S:GLU 536[OE2]	3.0
	H:TYR 58[OH]	S:LYS 496[NZ]	3.0
	H:TYR 58[OH]	S:TRP 535[O]	3.2
	H:GLY 96[O]	S:TRP 535[NE1]	3.0
	H:ASN 97[OD1]	S:LYS 543[NZ]	2.9
	H:ASN 97[ND2]	S:SER 528[O]	2.8
	H:SER 98[OG]	S:LYS 543[NZ]	3.7
	L:ASP 28[OD2]	S:LYS 400[NZ]	2.7
	L:ASN 30[ND2]	S:ASN 398[O]	3.0
	L:TYR 32[OH]	S:ASN 398[ND2]	2.7
	L:ALA 91[O]	S:SER 532[OG]	3.3
	L:ASN 92[O]	S:SER 532[OG]	2.3
Salt bridges			
	H:ASP 101[OD2]	S:LYS 543[NZ]	3.7
	L:ASP 28[OD1]	S:LYS 400[NZ]	3.9
	L:ASP 28[OD2]	S:LYS 400[NZ]	2.7

Supplementary Table 2b. Buried surface area of D12 antibody in complex with MERS England1 RBD. Residues that form hydrogen bonds and salt bridges are indicated.

D12 antibody

Residue	Bond type	Accessible Surface Area (Å²)	Buried Surface Area (Å²)
H:PHE 27		34.2	3.8
H:THR 28		84.5	7.0
H:SER 31		73.5	23.9
H:TYR 32	H	70.1	45.9
H:ALA 33		26.1	19.8
H:TRP 47		86.5	2.3
H:THR 50		24.4	21.9
H:SER 52	H	26.9	26.9
H:SER 52a	H	42.8	28.9
H:GLY 53	H	54.1	8.9
H:THR 55	H	104.7	14.7
H:TYR 56		132.7	39.2
H:TYR 58	H	116.7	79.7
H:ASP 95		15.4	6.6
H:GLY 96	H	59.3	47.8
H:ASN 97	H	153.7	88.1
H:SER 98	H	61.2	8.6
H:ASP 101	HS	62.4	18.7
L:GLN 27		103.7	15.4
L:ASP 28	HS	94.0	30.9
L:ASN 30	H	90.0	61.7
L:TYR 32	H	86.0	75.5
L:LEU 46		46.8	5.4
L:TYR 49		95.5	67.2
L:TYR 50		84.2	52.7
L:ARG 53		125.4	36.4
L:LEU 54		69.5	15.3
L:ASP 55		72.0	9.4
L:SER 56		96.9	31.6
L:GLY 68		26.4	2.7
L:ALA 91	H	46.3	10.1
L:LEU 92	H	52.0	21.3
L:SER 93		65.8	21.9
L:SER 94		159.6	57.3
L:SER 96		64.1	1.5

MERS England1 RBD

Residue	Bond type	Accessible Surface Area (Å²)	Buried Surface Area (Å²)
S:GLY 391		35.0	12.3
S:THR 392		77.1	23.8
S:PRO 394		28.8	7.9
S:TYR 397		1.3	1.3
S:ASN 398	H	41.1	34.8
S:PHE 399		4.0	2.3
S:LYS 400	HS	103.5	51.7
S:LEU 495		161.0	56.8
S:LYS 496	H	49.1	28.4
S:PRO 525		58.0	2.3
S:VAL 527		52.9	41.3
S:SER 528	H	88.4	88.4
S:ILE 529		17.5	14.1
S:VAL 530		11.1	11.1
S:PRO 531		35.5	30.3
S:SER 532	H	86.8	75.0
S:THR 533		39.8	0.4
S:TRP 535	H	193.7	178.1

S:GLU 536	H	116.2	89.7
S:ASP 537		83.0	4.2
S:ASP 539	H	36.8	26.6
S:TYR 540		161.9	22.2
S:TYR 541		29.9	11.7
S:ARG 542	H	136.8	50.7
S:LYS 543	H	121.9	115.9
S:GLN 544		122.4	35.0
S:LEU 545		32.6	2.8
S:SER 546		48.9	13.2
S:GLU 549		102.2	9.6
S:TRP 553		68.8	0.6
S:THR 560		33.1	2.7

H: Hydrogen bond; S: Salt bridge

Supplementary References

1. Prabakaran, P., *et al.* Structure of severe acute respiratory syndrome coronavirus receptor-binding domain complexed with neutralizing antibody. *The Journal of biological chemistry* **281**, 15829-15836 (2006).
2. Zhu, Z., *et al.* Potent cross-reactive neutralization of SARS coronavirus isolates by human monoclonal antibodies. *Proceedings of the National Academy of Sciences of the United States of America* **104**, 12123-12128 (2007).
3. Pak, J.E., *et al.* Structural insights into immune recognition of the severe acute respiratory syndrome coronavirus S protein receptor binding domain. *Journal of molecular biology* **388**, 815-823 (2009).
4. Hwang, W.C., *et al.* Structural basis of neutralization by a human anti-severe acute respiratory syndrome spike protein antibody, 80R. *The Journal of biological chemistry* **281**, 34610-34616 (2006).
